# Synthesis and Investigation of the G-Quadruplex Binding Properties of Kynurenic Acid Derivatives with a Dihydroimidazoquinoline-3,5-dione Core

**DOI:** 10.3390/molecules27092791

**Published:** 2022-04-27

**Authors:** Stefania Mazzini, Salvatore Princiotto, Loana Musso, Daniele Passarella, Giovanni Luca Beretta, Paola Perego, Sabrina Dallavalle

**Affiliations:** 1 Department of Food, Environmental and Nutritional Sciences (DeFENS), University of Milan, Via Celoria 2, 20133 Milan, Italy; stefania.mazzini@unimi.it (S.M.); loana.musso@unimi.it (L.M.); sabrina.dallavalle@unimi.it (S.D.); 2 Department of Chemistry, University of Milan, 20133 Milan, Italy; daniele.passarella@unimi.it; 3 Molecular Pharmacology Unit, Department of Applied Research and Technological Development, Fondazione IRCCS Istituto Nazionale Tumori, Via Amadeo 42, 20133 Milan, Italy; giovanni.beretta@istitutotumori.mi.it (G.L.B.); paola.perego@istitutotumori.mi.it (P.P.)

**Keywords:** kynurenic acid, KYNA, G-quadruplex, DNA, stabilization, cytotoxicity

## Abstract

G-quadruplexes are secondary structures originating from nucleic acid regions rich in guanines, which are well known for their involvement in gene transcription and regulation and DNA damage repair. In recent studies from our group, kynurenic acid (KYNA) derivative **1** was synthesized and found to share the structural features typical of G-quadruplex binders. Herein, structural modifications were conducted on this scaffold in order to assist the binding with a G-quadruplex, by introducing charged hydrophilic groups. The antiproliferative activity of the new analogues was evaluated on an IGROV-1 human ovarian cancer cell line, and the most active compound, compound **9,** was analyzed with NMR spectrometry in order to investigate its binding mode with DNA. The results indicated that a weak, non-specific interaction was set with duplex nucleotides; on the other hand, titration in the presence of a G-quadruplex from human telomere d(TTAGGGT)_4_ showed a stable, although not strong, interaction at the 3′-end of the nucleotidic sequence, efficiently assisted by salt bridges between the quaternary nitrogen and the external phosphate groups. Overall, this work can be considered a platform for the development of a new class of potential G-quadruplex stabilizing molecules, confirming the crucial role of a planar system and the ability of charged nitrogen-containing groups to facilitate the binding to G-quadruplex grooves and loops.

## 1. Introduction

**Figure 1:**
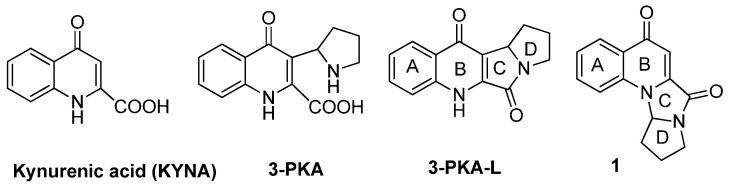
Structures of KYNA, its metabolites 3-PKA and 3-PKA-L and the analogue **1**.

The kynurenine pathway, leading to kynurenic acid (KYNA, Figure 1) together with several other biologically active metabolites, is the principal route for the catabolism of tryptophan [1]. Extensive studies concerning the biological role of KYNA revealed its neuroprotective and anticonvulsant properties based on receptor interactions [2,3]. Other investigations indicated the anti-inflammatory [4,5], analgesic [2,6,7], anti-ulcerative [8,9,10], antiatherogenic [11], antioxidative [12] and hepatoprotective [13] properties of KYNA. Several studies focusing on the potential role of KYNA in carcinogenesis and cancer therapy indicated that KYNA in millimolar concentrations exerts antiproliferative activity towards several cancer cell lines such as human glioblastoma T98G cells [14], human colon and renal cancer cells [15,16], colon adenocarcinoma HT-29, LS-180 and Caco-2 cells [15]. Compatible with these findings are reports on the action of KYNA on cell cycle regulators and signaling pathways involved in cell proliferation [16,17,18] and on transporters responsible for drug resistance in cancer, such as MRP4 [19] and BCRP [20]. Furthermore, it has been found that KYNA potentiates the effectiveness of cytostatic drugs [14] used in the therapy of cancer. The molecular mechanisms of the biological activity of KYNA in cancer cells have not been fully elucidated, and further studies should be undertaken. However, the interest towards this molecule and its analogues and/or metabolites is growing rapidly, due to its potential beneficial effects. Among KYNA derivatives, 3-PKA (Figure 1) represents the first example of a hybrid quinoline/pyrrolidine alkaloid found in chestnut honey in a wide range of concentrations [21]. The 3-PKA open form slowly undergoes conversion to its stable tetracyclic gamma-lactam form (3-PKA-L, Figure 1) through an intramolecular dehydration mechanism [22]. With the purpose of investigating the potential antitumor activity of KYNA derivatives, we undertook the synthesis of 3-PKA-L [23] and found that the compound had cytotoxic activity against human CRPC cells (castration-resistant prostate cancer) in the mM range [22]. These results prompted us to improve 3-PKA-L pharmacological potency through targeted chemical modification approaches. A few years ago, we developed a reliable synthetic route to a series of 4-quinolone-based compounds containing the fused tetracyclic system of compound **1** (Figure 1) [24]. Intrigued by the structural similarity between compound **1** and 3-PKA-L (same A-D rings, but different interconnection between the ring B and the ring C), we envisaged that compound **1** could be considered an interesting starting point for the development of new 3-PKA-L analogues and we were encouraged to channel our efforts toward this new scaffold.

In recent years, we have concentrated our research activity on the investigation of potential DNA stabilizing agents. [25,26,27]. In this context, particular attention was focused on G-quadruplex, a non-canonical secondary structure of nucleic acids present in the guanine-rich sequences of genomes [28]. G-quadruplexes are involved in the regulation of replication [29], DNA damage repair [30,31] and the transcription of oncogenes or other cancer-related genes [32,33,34,35]. Therefore, targeting G-quadruplexes has become a novel promising antitumor strategy. There are several common structural characters of G-quadruplex ligands, including a polycyclic core that can be combined with G-quadruplexes [36], and some charged hydrophilic groups to facilitate binding to G-quadruplex grooves and loops [37]. Based on the above characteristics, we envisaged that compound **1** shared certain structural features with known quadruplex-binding small molecules, such as the potential for a flat conformation, but it lacked charged groups which could eventually increase the binding with G-quadruplex [38]. With this in mind, our initial efforts were directed to the introduction of amino groups connected by proper spacers to the tetracyclic core.

In this paper, we report the results of our studies aimed at preparing analogues of compound **1** and investigating their potential interaction with DNA through NMR spectroscopy.

## 2. Results and Discussion

### 2.1. Synthesis

**Scheme 1:**
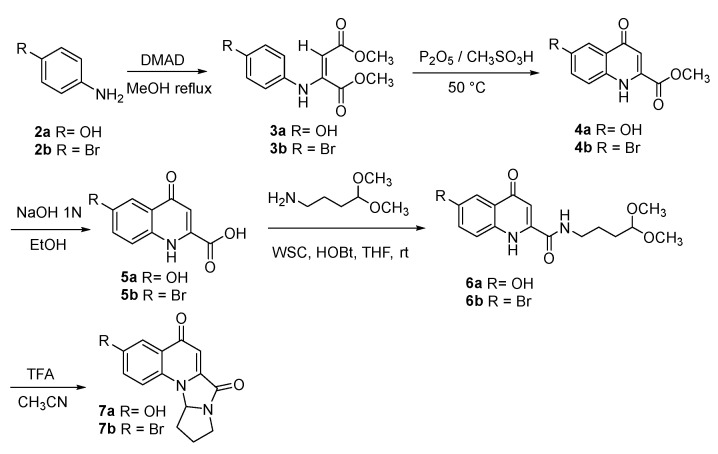
Synthesis of compounds **7a,b**.

Functionalized analogues of compound **1**, containing a phenolic –OH or a Br group on carbon 6, were prepared as described in Scheme 1.

4-Aminophenol (**2a**) was reacted with dimethylacetylene dicarboxylate, giving compound **3a**, which was cyclized to obtain compound **4a** with Eaton reagent (7.7% solution of P_2_O_5_ in methanesulfonic acid). Hydrolysis by NaOH 1 N, followed by reaction with 4-aminobutyraldehyde dimethyl acetal in the presence of WSC and HOBt, gave compound **6a**, which was then treated with trifluoroacetic acid. Tandem hetero-annulation allowed for the formation of compound **7a** [24].

**Scheme 2:**
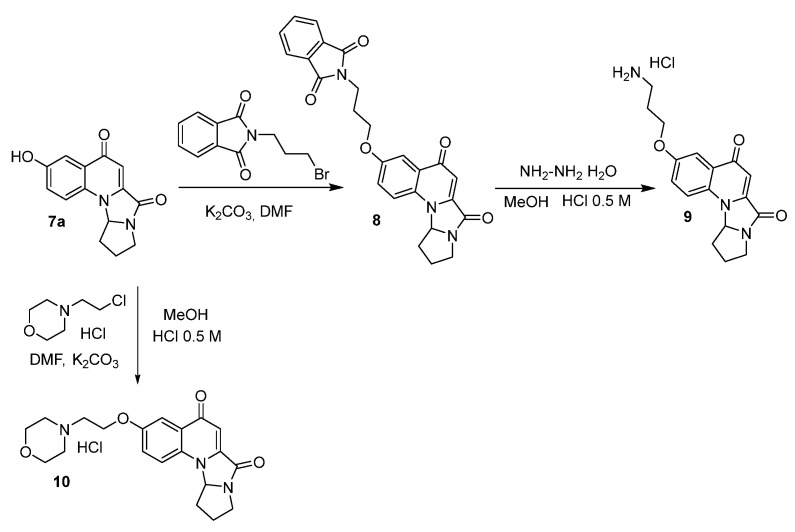
Synthesis of compounds **9** and **10**.

Compound **7a** was reacted with N-(3-bromopropyl)phtalimide to give compound **8**. Treatment with hydrazine afforded the free amine, which was suspended in 0.5 M HCl to obtain the hydrochloric salt **9**. Derivative **10** was obtained after treatment of compound **7a** with 4-(2-chloroethyl)morpholine hydrochloride, followed by hydrochloric acid addition (Scheme 2).

**Scheme 3:**
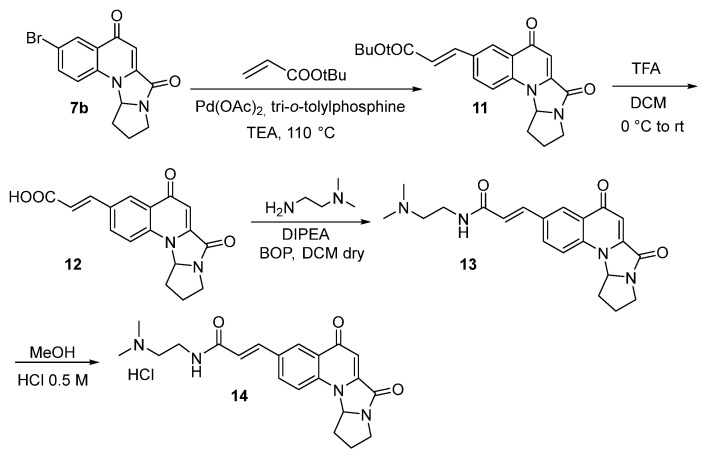
Synthesis of compound **14**.

Heck reaction between intermediate **7b** and tert-butylacrilate gave compound **11**, which after TFA treatment gave compound **12**. Coupling with N,N-dimethyl ethylenediamine in the presence of BOP and DIPEA afforded acrylamide **13**, which was then treated with 0.5 M HCl to obtain the salt **14** (Scheme 3).

### 2.2. Biological Activity

The antiproliferative activity of compounds **9**, **10** and **14** was evaluated on human tumor cell lines, including ovarian carcinoma IGROV-1 and prostate cancer CW22Rv1 cells, and on healthy RWPE1 prostate cells. Cytotoxicity was assessed with a growth inhibition assay after 72 h drug exposure. The results show that compound **9**, which contains the 3-aminopropoxy chain, was active on IGROV-1 cells (IC_50_ = 14.5 µM), while it was not active on prostate tumor CW22Rv1 and normal RWPE1 cells (IC_50_ > 100 µM). Compounds **10** and **14** showed no cytotoxicity (IC_50_ > 30–100 µM) on all the cells considered (Table 1). These findings indicate that the 3-aminopropoxy residue plays a role in antiproliferative potency against IGROV-1 cells. Thus, compound **9** was selected for studies of interaction with DNA through NMR spectroscopy.

### 2.3. Mode of Binding of Compound ***9*** with Duplex and G-Quadruplex Structures by NMR Spectroscopy

**Figure 2:**
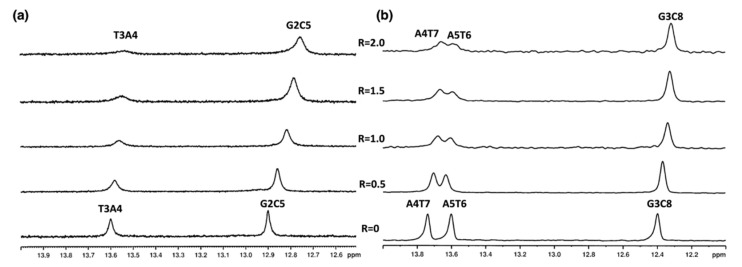
Imino proton region of the ^1^H NMR titration spectra of (**a**) 5′-d(CGTACG)_2_-3′ and (**b**) 5′d-(AAGAATTCTT)_2_-3′ with compound **9** at 15 °C in H_2_O/D_2_O (9:1), 100 mM NaCl and 10 mM sodium phosphate buffer (pH 7.0), at different ratios R = [9]/[DNA].

We initially investigated changes in the DNA double helix upon compound **9** binding. Titration experiments with both 5′-d(CGTACG)_2_-3′ and 5′-d(AAGAATTCTT)_2_-3′ oligonucleotide solutions were performed. In the presence of compound **9,** NH imino protons were slightly upfield shifted and underwent a broadening (Figure 2a,b and Table S1).

**Figure 3:**
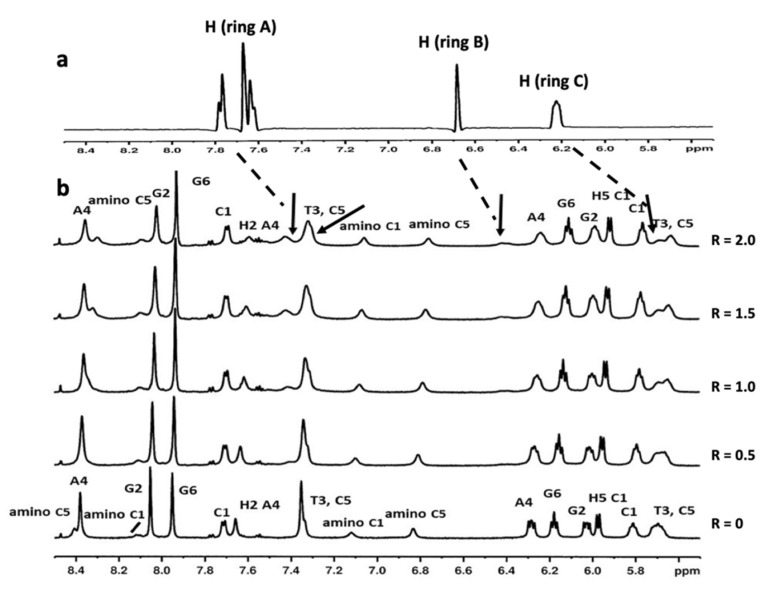
(**a**) Selected ^1^H proton region of compound **9** at 15 °C in H_2_O/D_2_O (9:1), 100 mM NaCl and 10 mM sodium phosphate buffer (pH 7.0); (**b**) aromatic and anomeric proton region of the ^1^H NMR titration spectra of 5′-d(CGTACG)_2_-3′ with compound **9** at 15 °C in H_2_O/D_2_O (9:1), 100 mM NaCl and 10 mM sodium phosphate buffer (pH 7.0), at different ratios R = [9]/[DNA]. The arrows indicate the broad proton signals of compound **9**.

**Figure 4:**
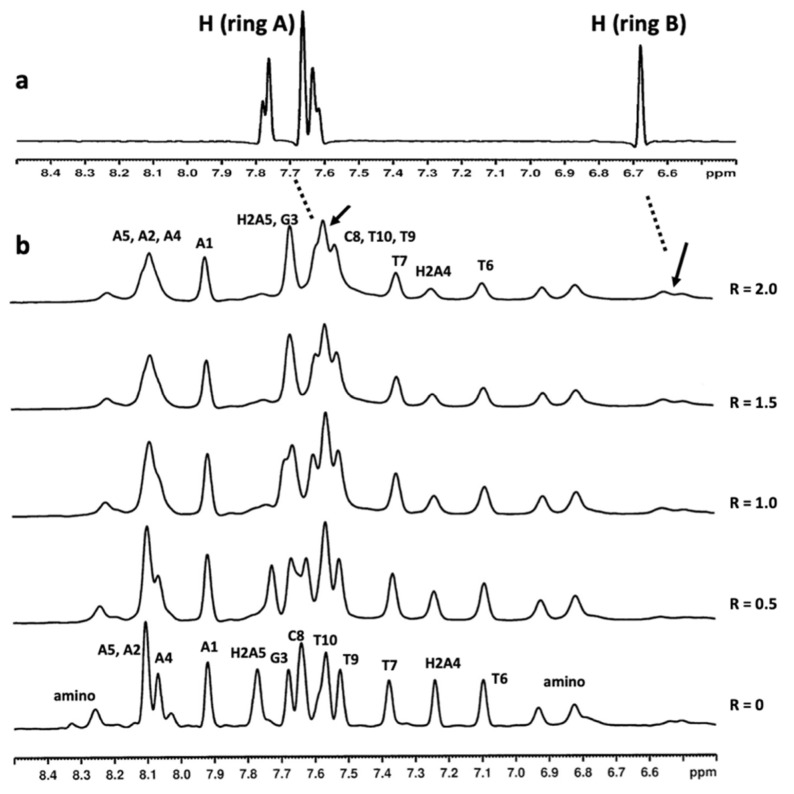
(**a**) Selected ^1^H proton region of compound **9** at 15 °C in H_2_O/D_2_O (9:1), 100 mM NaCl and 10 mM sodium phosphate buffer (pH 7.0); (**b**) aromatic proton region of the ^1^H NMR titration spectra of 5′d-(AAGAATTCTT)_2_-3′ with compound **9** at 15 °C in H_2_O/D_2_O (9:1), 100 mM NaCl and 10 mM sodium phosphate buffer (pH 7.0), at different ratios R = [9]/[DNA]. The arrows indicate the broad proton signals of compound **9**.

In particular, the T3A4 imino proton of 5′-d(CGTACG)_2_-3′, A4T7 and the A5T6 imino protons of 5′-d(AAGAATTCTT)_2_-3′ broadened significantly at R = [9]/[DNA] ratio = 2.0. The decrease in their intensity can be interpreted as the effect of the weakening of the hydrogen bonds associated with a dynamic process between free and complexed oligonucleotides. Changes in the line broadening of the aromatic and anomeric protons in the complex with compound **9** were also observed for both oligonucleotides. The lower intensity of some signals of compound **9** at higher [ligand]/[DNA] ratios can also be explained by a mobility of the drug in the binding site and by free-ligand-bound ligand exchanges (Figure 3 and Figure 4).

The most affected signals belonged to the central residues of the following sequences: H6 and H1′T3, H2 and H1′ A4 of 5′-d(CGTACG)_2_-3′; and H2 A4, H2 A5, H6T6 and H6 T7 of 5′-d(AAGAATTCTT)_2_-3′. Moreover, aromatic protons of G3, C8 T9 and T10 units collapsed together between δ 7.69 and δ 7.50 ppm (Figure 4). The signals of compound **9** at δ 7.42 and δ 6.40 ppm in the 5′-d(CGTACG)_2_-3′/**9** complex, and at δ 6.53, δ 6.48 and between δ 7.69 and δ 7.50 ppm in the 5′-d(AAGAATTCTT)_2_-3′/**9** complex, assigned by the 2D NOESY and TOCSY experiments, were too broad to identify intermolecular NOE interactions.

As reported in the literature [39], there are three major modes of binding to DNA: (1) intercalation, (2) outside binding in groove and (3) nonspecific outside binding. For the majority of intercalating drugs, large upfield shifts in both the A-T and the G-C resonances have been observed, often accompanied by some broadening of the resonance. For the class of compounds working as outside binders in a groove, downfield shifts of the A-T and to a smaller extent the G-C resonances have been reported. Eventually, nonspecific outside binders give very little or no spectral changes in the low-field spectral region. For this group of drugs, binding to the outside of the DNA in an unspecified manner (possibly through electrostatic interactions with the phosphate backbone) is proposed. In our case, the addition of compound **9** to either of the two duplexes was coupled with broadening and slight (below 0.1 ppm) perturbations in the imino ^1^H NMR signals belonging to AT base pairs. These findings, supported by our experience in the field [40,41], allowed us to exclude an intercalation of compound **9** with either of the two oligonucleotides, but suggested a slight association through an external binding mode at the level of AT base pairs.

We then focused our attention on the G-quadruplex binding properties of compound **9** towards G-quadruplex structures.

The first biologically relevant G-quadruplex-forming sequences were observed in telomeric DNA [42]. The formation of telomere–G-quadruplex as been shown to inhibit the telomerase activity, a reverse transcriptase that extends the telomeric sequence at the chromosome ends, preventing critical shortening of the telomeric DNA [43]. We therefore used the single repeat sequence of human telomeres, d(TTAGGGT)_4_, as a model G-quadruplex structure to investigate the potential G-quadruplex-targeting ability of compound **9**.

**Figure 5:**
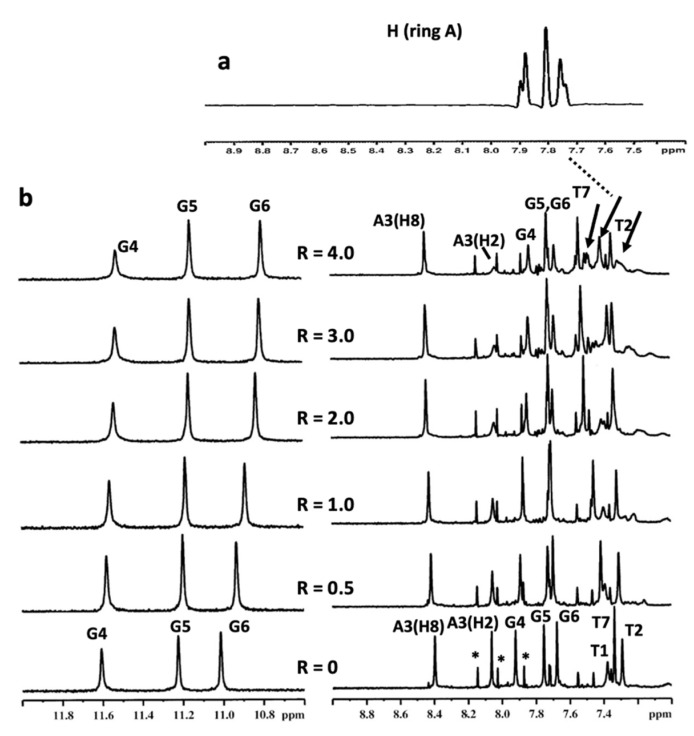
(**a**) Selected region of ^1^H NMR spectrum of compound **9** at 25 °C in H_2_O/D_2_O (9:1), 150 mM KCl, 25 mM K^+^ phosphate buffer and EDTA 1 mM (pH 6.7); (**b**) imino and aromatic proton region of the ^1^H NMR titration spectra of d(TTAGGGT)_4_ with compound **9** at 25 °C in H_2_O/D_2_O (9:1), 150 mM KCl, 25 mM K^+^ phosphate buffer and EDTA 1 mM (pH 6.7), at different ratios R = [9]/[DNA]. The arrows indicate the broad proton signals of compound **9**. The peaks labelled with * correspond to the single-strand oligonucleotide.

The d(TTAGGGT)_4_ was titrated up to a 4:1 (ligand/G-quadruplex) stoichiometry (Figure 5). In 2D NOESY experiments, the observed NOEs between adjacent protons such as NH-NH, aromatic and NH-MeT indicated that the three tetrads were intact in the complex. In order to evaluate the specific oligonucleotide residues involved in the interaction with compound **9**, the Δδ values of the NH imino, aromatic, anomeric and methyl proton resonance of the complex in comparison with the free oligonucleotide were calculated (Table S2).

The titration of a ligand into d(TTAGGGT)_4_ can result in significant line broadening which can be attributed to the intermediate exchange of the drug between a number of possible bound conformations and/or a number of sites of similar affinity. Usually, the NH imino protons of G4 and G6 are appreciably shifted by up to 0.8 ppm [26,44,45,46].

In our experiments, the G6 NH imino proton had an upfield shift of 0.21 ppm, while the aromatic and methyl protons of the T7 unit moved downfield (0.20 and 0.15 ppm, respectively). All the other protons of the oligonucleotide did not show a significant change in chemical shift values upon increasing the ligand concentration (NH imino protons of G4 and G5 shifted upfield by 0.09 and 0.07 ppm, respectively). Moreover, the upfield shifts of the A3(H2), G4 and G5 aromatic protons were in the range of 0.08 and 0.05 ppm. Moreover, some signals of compound **9** experienced a chemical shift variation and a notable broadening (Figure 5). Interestingly, G4 NH showed line broadening; however, it underwent a small upfield chemical shift variation (0.09 ppm). A possible explanation of this behavior could be related to the exchange of imino protons involved in the interstrand hydrogen bonds, as a consequence of an increased flexibility caused by the ligand interaction with G6 and the groove of the quadruplex. This could lead first to broadening, and then to the disappearance of the imino signals. These findings, in line with previous results in the field [26], suggest that compound **9** binds the G-quadruplex in an intermediate regime on the NMR time scale. Furthermore, the compound is able to interact with the aromatic ring at the 3′-end tetrad (G6), but is unable to provide efficient stacking, most likely due to the poor extension of the aromatic system. The rest of the molecule can extend in the groove/side of DNA without perturbation of the central (G5) and 5′-end (G4) tetrads, excluding an intercalation binding mode. Indeed, the absence of NOEs between compound **9** and the G-quadruplex should be an important clue that there might be several different binding sites with similar binding affinities, perhaps requiring compound **9** to adopt different conformations, altogether contributing to the broadness of the ^1^H NMR signals.

**Figure 6:**
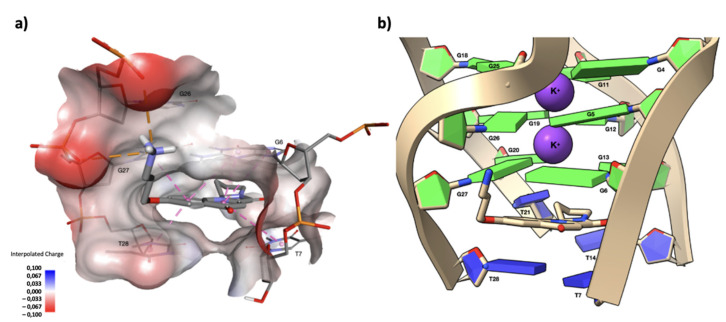
(**a**) Drawing that shows the first interaction shell of compound **9** complexed with the d(TTAGGGT)_4_ G-quadruplex, as obtained from the molecular docking experiment. In this position, compound **9** is capable of forming multiple π–π stacking interactions between the aromatic rings of the polycycle and those of the G6 and T7 units. The side chain of compound **9** orients along the groove, with the quaternary nitrogen forming two salt bridges with G5OP_2_ and G6OP_2_, contributing to the stabilization of the complex. In the figure, G5, G6 and T7 are labelled as G26, G27 and T28, respectively, due to the loss of symmetry in the complex. The non-bonded interactions are displayed as dashed lines, the π–π stacking interactions in pink, and the two salt bridge interactions in orange. A surface near the current ligand is created around the target and colored by the interpolated atomic charge of the d(TTAGGGT)_4_ G-quadruplex atoms. Nucleotides and the ligand are represented in sticks and colored according to their atom types. (**b**) A close-up view of the capping at the ligands interacting with the 5′-end tetrad. Nucleotide bases are represented as slabs and fill sugars, with the guanine residues colored in green and thymine residues colored in blue. Potassium ions are represented as purple van der Waals spheres, while the ligand is drawn in stick and colored according to its atom types. The drawing was created using the Discovery Studio^®^ Visualizer (BIOVIA, Dassault Systèmes Discovery Studio Modeling Environment, Release 2017) and UCSF ChimeraX, developed by the Resource for Biocomputing, Visualization, and Informatics at the University of California, San Francisco, with support from the National Institutes of Health R01-GM129325 and the Office of Cyber Infrastructure and Computational Biology, National Institute of Allergy and Infectious Diseases.

The three-dimensional model of the top ranked complex between compound **9** and the d(TTAGGGT)_4_ parallel-stranded DNA G-quadruplex, as obtained from the molecular docking experiment, is shown in Figure 6.

The ligand fits between the G6 tetrad and T7 of the d(TTAGGGT)_4_ G-quadruplex. In this position, compound **9** is capable of forming multiple π–π stacking interactions between the aromatic rings of the polycycle and those of the G6 and T7 units. However, our experimental results (G6 NH Δ δ = −0.21 ppm, see above) demonstrated that this π–π stacking interaction is scarcely efficient, most likely due to the limited extension of the aromatic system (see above). The side chain of compound **9** orients along the groove, with the quaternary nitrogen forming two salt bridges with G5OP_2_ and G6OP_2_, contributing to the stabilization of the complex.

However, it is important to note that the binding of compound **9** to the external tetrad proposed by the docking experiments is not the sole possible interpretation of the experimental results; in fact, it may represent one among several possible binding modes.

## 3. Material and Methods

### 3.1. Chemistry

All reagents and solvents were reagent grade or were purified by standard methods before use. Melting points were determined in open capillaries with an SMP3 apparatus and were uncorrected. ^1^H spectra were recorded on Bruker AMX 300 and Bruker AV600 spectrometers (Karlsruhe, Germany). TMS was used as an internal standard and the chemical shifts were reported in parts per million (δ). The peak patterns are indicated as follows: s, singlet; d, doublet; dd, doublet of doublet; t, triplet; m, multiplet; q, quartet. The coupling constants, J, are reported in Hertz (Hz).

The solvents were routinely distilled prior to use; anhydrous tetrahydrofuran (THF) and ether (Et_2_O) were obtained by distillation from sodium-benzophenone ketyl; dry methylene chloride was obtained by distillation from phosphorus pentoxide. All reactions requiring anhydrous conditions were performed under a positive nitrogen flow and all glassware were oven dried. The isolation and purification of the compounds were performed with flash column chromatography on silica gel 60 (230–400 mesh). Analytical thin-layer chromatography (TLC) was conducted on TLC plates (silica gel 60 F254, aluminum foil). Compounds on the TLC plates were detected under UV light at 254 and 365 nm or were revealed by spraying with 10% phosphomolybdic acid (PMA) in ethanol. All the chemicals were purchased from Sigma-Aldrich (St. Louis, Missouri, United States).

The analyses indicated by the symbols of the elements or functions were within ±0.4% of the theoretical values.

Compounds **3b**, **4b** and **5b** were prepared according to the standard procedures in the literature [24], as was *N*-(3-bromopropyl)phtalimide [47].

2-(4-Hydroxyphenylamino)-but-2-enedioic acid dimethyl ester (**3a**)

To a solution of 4-aminophenol (1.5 g, 13.74 mmol) in CH_3_OH (44 mL), DMAD (1.7 mL, 13.74 mmol) was added dropwise. The reaction mixture was stirred for 2 h at reflux; and was then cooled at room temperature before the solvent was evaporated. The crude residue was purified through flash column chromatography (ETP/AcOEt 65:35) to give compound **3a** as a yellow oil (2.31 g, 84%). R*_f_* (ETP/AcOEt 1:1) = 0.81; ^1^H NMR (300 MHz, CDCl_3_) *δ*: 9.51 (1H, s); 6.89–6.78 (4H, m); 5.26 (1H, s); 6.72 (3H, s); 6.67 (1H, s).

6-Hydroxy-4-oxo-1,4-dihydroquinoline-2-carboxylic acid methyl ester (**4a**)

Eaton reagent (4.6 mL) was added to 3a (1.09 g, 4.34 mmol) under nitrogen and the mixture was stirred and heated for 1h at 55 °C. The reaction was cooled at room temperature, poured into a Na_2_CO_3_ saturated solution (46 mL), then cooled at 0 °C. The precipitate was filtered under vacuum and washed with water to obtain compound **4a** as a white solid (657 mg, 70%). R*_f_* (Hex/AcOEt 6:4) = 0.3; m.p. 279 °C; ^1^H NMR (600 MHz, DMSO-*d*_6_) *δ*: 12.05 (1H, bs); 9.98 (1H, brs), 7.81 (1H, d, J = 8.6 Hz), 7.36 (1H, d, J = 2.2 Hz), 7.21 (1H, dd, J =2.2, 8.6 Hz); 6.55 (1H, s); 3.92 (3H, s); ^13^C NMR (150 MHz, DMSO-*d6*) δ: 176.6, 163.2, 154.7, 137.0, 133.9, 127.2, 123.1 (×2C), 107.8, 106.5, 53.2.

6-Hydroxy-4-oxo-1,4-dihydroquinoline-2-carboxylic acid (**5a**)

To a suspension of compound **4a** (652 mg, 3.0 mmol) in CH_3_OH (10 mL), 1N NaOH (10 mL) was added. The resulting mixture was heated at reflux for 1 h 30**′**, then CH_3_OH was evaporated. After cooling with an ice bath, 1N HCl was added and the white solid formed was filtered under vacuum and dried to give compound **5a** (484 mg, 79%). R*_f_* (DMC/CH_3_OH 8:2) = 0.1; m.p. 299 °C (dec.); ^1^H-NMR (600 MHz, DMSO-*d*_6_), δ: 12.01 (1H, bs), 9.92 (1H, brs), 7.82 (1H, d, J = 8.7 Hz), 7.34 (1H, d, J = 2.3 Hz), 7.20 (1H, dd, J =2.3, 8.7 Hz); 6.59 (1H, s); ^13^C NMR (150 MHz, DMSO-*d*_6_) δ: 176.0, 163.9, 154.4, 138.3, 133.9, 127.1, 123.0, 121.7, 107.7, 106.6.

6-Hydroxy-4-oxo-1,4-dihydroquinoline-2-carboxylic acid (4,4-dimethoxybutyl)-amide (**6a**)

To a suspension of compound **5a** (200 mg, 0.98 mmol) in THF (7 mL) under nitrogen, WSC (281 mg, 1.46 mmol), HOBt (198 mg, 1.46 mmol) and 4-aminobutyraldehyde dimethyl acetal (180 μL, 1.46 mmol) were added sequentially. The mixture was heated at reflux for 3 h. The solvent was evaporated, water was added and the solid was filtered under vacuum. The crude was purified through flash chromatography (DCM/CH_3_OH 95:5 to 9:1) to give compound **6a** as a white solid (181 mg, 58%). R*_f_* (DCM/CH_3_OH 8:2) = 0.61; m.p.> 289 °C (dec.); ^1^H-NMR (600 MHz, DMSO-*d*_6_), δ: 11.69 (1H, bs); 9.75 (1H, s), 8.91 (1H, t, m); 7.81 (1H, d, J = 8.7 Hz), 7.32 (1H, d, J = 2.3 Hz), 7.16 (1H, m); 6.57 (1H, s); 4.29–4.37 (1H, m); 3.42–3.21 (2H, m); 3.20 (6H, s); 1.62–1.41 (4H, m); ^13^C NMR (150 MHz, DMSO-*d_6_*) δ: 177.0, 162.1, 154.1, 137.1, 133.4, 127.1, 122.6, 121.1, 106.8, 103.7 (× 2C), 52.4, 1C missing due to the overlap with solvent signal, 29.6, 24.1.

3-Hydroxy-8,9,10,10a-tetrahydro-7a,10b-diazapentaleno[1,2-a]naphthalene-5,7-dione (**7a**)

Trifluoroacetic acid (145 μL, 0.850 mmol) was added dropwise to a suspension of compound 6a (180 mg, 0.566 mmol) in CH_3_CN (10 mL). The mixture was heated at reflux for 1 h. The solvent was evaporated, and the residue was triturated with Et_2_O and filtered under vacuum, obtaining compound **9a** (135 mg, 93%). R*_f_* (DCM/CH_3_OH 95:5) = 0.45; m.p. 303 °C (dec.); ^1^H-NMR (600 MHz, DMSO-*d*_6_), δ: 10.01 (1H, bs); 7.62 (1H, d, J = 8.5 Hz); 7.51 (1H, d, J = 2.1 Hz); 7.30 (1H, dd, J = 2.1, 8.5 Hz); 6.28 (1H, s); 6.14–5.92 (1H, m); 3.78–3.53 (1H, m); 2.88–2.62 (1H, m); 2.41–2.11 (2H, m); 1.78–1.50 (1H, m). ^13^C NMR (150 MHz, DMSO-*d*_6_) δ 175.9, 163.6, 155.3, 141.7, 130.4, 127.5, 123.2, 118.2, 116.3, 114.4, 108.6, 100.7, 77.8, 41.6, 29.5, 25.6.

4-(2-((5,7-dioxo-5,6,6a,7,9,10,11,11a-octahydropyrrolo[2′,1′:2,3]imidazo[1,5-a]quinolin-3-yl)oxy)ethyl)morpholin-4-ium chloride (**10**)

To a solution of compound **7a** (70 mg, 0.273 mmol) in dry DMF (2 mL), 4-(2-chloroethyl)-morpholine hydrochloride (56 mg, 0.301 mmol) and K_2_CO_3_ (162 mg, 1.174 mmol) were added under nitrogen. The reaction mixture was stirred and heated at reflux for 3 h. After TLC in DCM/CH_3_OH 9:1, an additional amount of 4-(2-chloroethyl)-morpholine hydrochloride (20 mg, 0.108 mmol) was added and the reaction was heated at reflux for 8 h. Additional amounts of 4-(2-chloroethyl) morpholine hydrochloride (50 mg, 0.273 mmol) and K_2_CO_3_ (88 mg, 0.273 mmol) were added, and the reaction was heated for 6 h. The solvent was then evaporated under vacuum and the crude compound purified through flash chromatography (DCM/CH_3_OH 93:7), obtaining 3-(2-Morpholin-4-yl-ethoxy)-8,9,10,10a-tetrahydro-7a,10b-diaza-pentaleno[1,2-a]naphthalene-5,7-dione as a yellow solid (80 mg, 79%). R*_f_* (DCM/CH_3_OH 9:1) = 0.51; m.p. 204.5 °C; ^1^H NMR (300 MHz, DMSO-*d*_6_) *δ*: 7.67 (1H, d, J = 8.7 Hz); 7.60 (1H, d, J = 3.2 Hz); 7.45 (1H, dd, J = 3.2, 8.7 Hz); 6.27 (1H, s); 6.10–6.00 (1H, m); 4.19 (2H, t, J = 5.7 Hz); 3.73–3.60 (1H, m); 3.59–3.53 (4H, m); 3.47–3.24 (6H, m); 2.72 (2H, t, J = 5.7 Hz); 2.38–2.14 (2H, m); 1.72–1.46 (1H, m); ^13^C NMR (75MHz, DMSO-*d*_6_) δ 177.3, 164.2, 156.3, 142.5, 132.1, 128.3, 124.1, 118.8, 107.7, 101.9, 78.2, 66.9 (×2C), 66.6, 57.5, 54.3 (×2C), 42.3, 30.2, 26.3.

To a suspension of the above compound (35 mg, 0.095 mmol) in CH_3_OH (2 mL) at 0 °C, 0.5 M HCl in CH_3_OH (237 μL, 0.095 mmol) was slowly added. The solution was stirred for 1h at room temperature, then the solvent was evaporated under vacuum and the solid obtained (36 mg, 94%) was dried. m.p. 292.4 °C; ^1^H-NMR (300 MHz, DMSO-*d*_6_), δ: 11.04 (1H, bs); 7.74 (1H, d, J = 8.5 Hz); 7.69 (1H, d, J = 2.8 Hz); 7.51 (1H, dd, J = 2.8, 8.5 Hz); 6.31 (1H, s); 6.12–6.01 (1H, m); 4.52 (2H, t, J = 5.6 Hz); 4.05–3.89 (5H, m); 3.88–3.69 (2H, m); 3.67–3.30 (4H, m); 3.28–3.05 (2H, m); 2.82–2.65 (1H, m); 2.37–2.14 (2H, m); 1.74–1.50 (1H, m).

3-[3-(1,3-Dioxo-1,3-dihydro-isoindol-2-yl)-propoxy]-8,9,10,10a-tetrahydro-7a,10b-diaza-pentaleno[1,2-a]naphthalene-5,7-dione (**8**)

To a solution of compound **7a** (300 mg, 1.172 mmol) in dry DMF (8.8 mL) under nitrogen, K_2_CO_3_ (858 mg, 6.212 mmol) and *N*-(3-bromopropyl)phtalimide (550 mg, 2.075 mmol) were added. The mixture was heated at reflux for 4 h. The solvent was evaporated, and the crude purified through flash chromatography (DCM/CH_3_OH 97.5:2.5) to give compound **8** as a light-yellow solid (435 mg, 84%). R*_f_* (DCM/CH_3_OH 95:5) = 0.46; m.p. 209 °C; ^1^H NMR (600 MHz, DMSO-*d*_6_) *δ*: 7.95-7.75 (4H, m); 7.65 (1H, d, J = 9.0 Hz); 7.55 (1H, d, J = 2.9 Hz); 7.29 (1H, dd, J = 2.9, 9.0 Hz); 6.28 (1H, s); 6.11-5.99 (1H, m);); 4.16 (2H, t, J = 5.9 Hz); 3.77 (2H, t, J = 6.4 Hz); 3.70–3.59 (1H, m); 2.68–2.71 (1H, m); 2.42–2.21 (2H, m); 2.0–2.21 (2H, m); 1.79–1.58 (1H, m); ^13^C-NMR (150MHz, DMSO-*d*_6_) δ: 176.6, 168.0 (×2C), 163.7, 155.8, 141.8, 134.3 (×2C), 131.8 (×2C), 131.5, 127.6, 123.0 (×3C), 117.9, 106.6, 101.5, 77.5, 66.0, 41.6, 34.9, 29.5, 27.5, 25.6.

3-((5,7-dioxo-5,7,9,10,11,11a-hexahydropyrrolo[2′,1′:2,3]imidazo[1,5-a]quinolin-3-yl)oxy)propan-1-aminium chloride (**9**)

To a solution of compound **8** (430 mg, 0.968 mmol) in CH_3_OH (6 mL), hydrazine hydrate (140 μL, 2.90 mmol) was added. The reaction mixture was heated at reflux for 5 h, then an additional amount of hydrazine hydrate (93 μL, 1.936 mmol) was added and the reaction was heated at reflux for 2 h. The solvent was evaporated and the solid obtained was purified through flash chromatography (DCM/CH_3_OH 9:1 with 2% of NH_3_), obtaining 3-(3-Amino-propoxy)-8,9,10,10a-tetrahydro-7a,10b-diazapentaleno[1,2-a]naphthalene-5,7-dione as a light yellow solid (156 mg, 51%). R_f_ (DCM/CH_3_OH 9:1 with 2% of NH_3_) = 0.14; m.p. 167.3 °C; ^1^H-NMR (300 MHz, CH_3_OH-*d*_4_) *δ*: 7.75 (1H, d, J = 2.9 Hz); 7.70 (1H, d, J = 9.3 Hz); 7.50 (1H, dd, J = 2.9, 9.3 Hz); 6.14–6.03 (1H, m); 4.19 (2H, t, J = 6.1 Hz); 3.89–3.76 (1H, m); 3.55–3.44 (1H, m); 2.97–2.77 (3H, m); 2.52–2.36 (2H, m); 2.09–1.96 (2H, m); 1.79–1.61 (1H, m); ^13^C-NMR (75 MHz, CH_3_OH-*d*_4_) δ: 180.2, 165.2, 158.4, 143.5, 133.1, 128.8, 125.6, 118.9, 107.4, 103.2, 79.7, 67.4, 42.9, 39.4, 32.4, 31.2, 26.9.

To a suspension of the above compound (50 mg, 0.095 mmol) in CH_3_OH (2 mL) at 0 °C, a solution of 0.5 M HCl in CH_3_OH (237 μL, 0.095 mmol) was slowly added. The solution was stirred at room temperature for 2 h; it was then evaporated under vacuum and the solid obtained (51 mg, 95%) was dried. m.p. 264.4 °C; ^1^H NMR (300 MHz, CH_3_OH-*d*_4_) *δ*: 7.82-7.71 (2H, m); 7.57 (1H, dd, J = 3.0, 9.3 Hz); 6.15 81H, dd, J = 5.4, 8.9 Hz); 4.26 (2H, t, J = 5.8 Hz); 3.90–3.76 (1H, m); 3.57–3.46 (1H, m); 3.21 82H, t, J = 5.8 Hz); 2.93–2.79 (1H, m); 2.54–2.38 (2H, m); 2.30–2.15 (2H, m); 1.81–1.60 (1H, m).

6-Bromo-4-oxo-1,4-dihydro-quinoline-2-carboxylic acid(4,4-dimethoxy-butyl)-amide (**6b**)

To a suspension of compound **5b** (437 mg, 1.77 mmol) in THF, WSC (510 mg, 2.66 mmol), HOBt (359 mg, 2.66 mmol) and 4-aminobutyraldehyde dimethyl acetal (373 μL, 2.66 mmol) were added sequentially under nitrogen. The reaction mixture was stirred over a weekend at room temperature. The solvent was evaporated, and the product was suspended in water, filtered and washed with a saturated solution of NaHCO_3_. Compound **8b** was obtained as a white solid (568 mg, 84%). R*_f_* (DCM/CH_3_OH 95:5) = 0.45; m.p. 192 °C; ^1^H-NMR (300 MHz, DMSO-d_6_), *δ*: 8.92-8.62 81H, m); 8.01 (1H, d, J = 1.5 Hz); 7.85–7.50 (2H, m); 6.78 (1H, s); 4.36–4.27 (1H, m); 3.26–2.96 (8H, m); 1.71–1.39 (4H, m).

3-Bromo-8,9,10,10a-tetrahydro-7a,10b-diazapentaleno[1,2-a]naphthalene-5,7-dione (**7b**)

Trifluoroacetic acid (170 μL, 2.22 mmol) was added dropwise to a suspension of compound **6b** (568 mg, 1.48 mmol) in CH_3_CN. The reaction mixture was heated at reflux for 3 h. After cooling, the mixture was evaporated and the sticky solid obtained was washed with Et_2_O three times, obtaining compound **7b** as a white/light grey solid (448 mg, 95%). R*_f_* (DCM/CH_3_OH 95:5) = 0.35; m.p. 263 °C; ^1^H NMR (600 MHz, DMSO-*d*_6_) *δ*: 8.27 (1H, d, J¼2.3 Hz), 7.98 (1H, dd, J = 2.3, 8.9 Hz), 7.72 (1H, d, J = 8.9 Hz), 6.37 (1H, s), 6.09–6.02 (1H, m), 3.73–3.63 (1H, m), 3.43–3.36 (1H, m), 2.79–2.68 (1H, m), 2.36–2.19 (2H, m), 1.73–1.60 (1H, m); ^13^C NMR (150 MHz, DMSO-*d*_6_) *δ*: 175.5, 162.6, 142.7, 135.4, 135.1, 127.7, 127.1, 118.4, 117.0, 102.7, 77.1, 41.1, 28.7, 25.0.

3-(5,7-Dioxo-5,6,6a,7,8,9,10,10a-octahydro-7a,10b-diaza-pentaleno[1,2-a]naphthalen-3-yl)-acrylic acid *tert*-butyl ester (**11**)

To a suspension of compound **7b** (300 mg, 0.940 mmol) in TEA (364 μL), Pd(OAc)_2_ (5 mg, 0.02 mmol), tert-butylacrilate (218 μL, 1.504 mmol) and tri-*o*-tolylphosphine (26 mg, 0.037 mmol) were added under nitrogen. The reaction was heated at reflux for 12 h. The mixture was then evaporated and dried under vacuum and the crude product was purified through flash chromatography (DCM/CH_3_OH 50:1 then 45:1) to obtain compound 11 (78 mg, 23%). R*_f_* (DCM/CH_3_OH 24:1) = 0.50; m.p. 280 °C; ^1^H-NMR (600 MHz, CDCl_3_), δ: 8.55 (1H, d, J = 1.8 Hz); 7.86 (1H, dd, J = 1.8, 8.4 Hz); 7.65 (1H, d, J = 16.3 Hz); 7.34 (1H, d, J = 8.4 Hz); 6.78 (1H, s); 6.48 (1H, d, J = 16.3 Hz); 5.84 (1H, dd, J = 5.3, 8.4 hz); 4.01–3.88 (1H, m); 3.56–3.42 (1H, m); 2.84–2.70 (1H, m); 2.57–2.29 (2H, m); 1.84–1.64 (1H, m); 1.54 (9H, s). ^13^C-NMR (150 MHz, CDCl_3_), δ: 178.5, 165.8, 163.7, 142.0, 141.6, 137.5, 131.7, 131.4, 127.7, 127.0, 121.6, 115.0, 106.0, 80.8, 41.9, 30.9, 28.2 (× 3C), 25.8.

3-(5,7-Dioxo-5,7,8,9,10,10a-hexahydro-7a,10b-diazapentaleno[1,2-a]naphthalen-3-yl)-acrylic acid (**12**)

To a suspension of compound **11** (73 mg, 0.2 mmol) in dry DCM (61 μL) at 0 °C, TFA (15 μL, 0.2 mmol) was added. After 10 min at 0 °C, the solution was stirred for 2 days at room temperature, then additional TFA (40 μL) was added and the solution was stirred at room temperature over the weekend. The reaction mixture was evaporated under vacuum and the solid residue was triturated with Et_2_O, obtaining 47.3 mg of compound **12** (76%). R*_f_* (DCM/CH_3_OH 24:1) = 0.14; m.p. 335 °C (dec); ^1^H-NMR (600 MHz, DMSO-*d*_6_), δ: 12.49 (1H, bs), 8.37 (1H, d, J = 2.0 Hz); 8.22 (1H, dd, J = 2.0, 8.8 Hz); 7.72 (1H, d, J = 8.8 Hz); 7.71 (1H, d, J = 15.9 Hz); 6.66 (1H, d, J = 15.9 Hz); 6.38 (1H, s); 6.11 (1H, dd, J = 5.4, 8.9 Hz); 3,74–3.60 (1H, s); 2.82–2.67 (1H, s); 2.35–2.19 (2H, m); 1.75–1.55 (1H, m); ^13^C-NMR (150 MHz, DMSO-*d_6_*), δ: 177.1, 167.4, 163.2, 142.9, 142.8, 137.8, 131.6, 130.6, 126.8, 126.3, 121.6, 120.0, 117.1, 103.5, 77.6, 41.6, 29.4, 28.2, 25.5.

N-(2-Dimethylamino-ethyl)-3-(5,7-dioxo-5,7,8,9,10,10a-hexahydro-7a,10b-diaza-pentaleno[1,2-a]naphthalen-3-yl)-acrylamide (**13**)

To a suspension of compound **12** (38 mg, 0.122 mmol) in dry DCM (1.5 mL) under nitrogen DIPEA (341 μL, 1.959 mmol), *N*,*N*-dimethylethylenediamine (14 μL, 0.129 mmol) and BOP (70 mg, 0.157 mmol) were added and the reaction was stirred for 48 h at room temperature. The solvent was evaporated under vacuum and the residue was triturated with Et_2_O and with Et_2_O/isopropanol (4.5:1), obtaining compound **13** (35.7 mg, 77%). R*_f_* (DCM/CH_3_OH 9:1) = 0.04; m.p. 236 °C; ^1^H NMR (300 MHz, DMSO-*d*_6_) *δ*: 9.39 (1H, brs); 8.53–8.28 (2H, m); 8.02 (1H, d, J = 8.5 Hz); 7.75 (1H, d, J = 8.5 Hz); 7.59 (1H, d, J = 15.7 Hz); 6.76 (1H, d, J = 15.7 Hz); 6.34 (1H, s), 6.06 (1H, m); 3.78–3.59 (1H, m); 3.60–3.50 (2H, m); 3.44–3.22 (2H, m); 3.23–3.13 (2H, m); 2.39–2.15 (2H, m); 1.78–1.56 (1H,m); ^13^C NMR (75 MHz, DMSO-*d*_6_) δ 177.2, 165.6, 163.2, 143.0, 138.1, 137.4, 132.0, 131.1, 126.4, 125.0, 122.4, 117.2, 103.3, 77.6, 56.2, 42.6, 41.5, 36.4, 39.4, 25.5.

(E)-2-(3-(5,7-dioxo-5,7,9,10,11,11a-hexahydropyrrolo[2′,1′:2,3]imidazo[1,5-a]quinolin-3-yl)acrylamido)-*N*,*N*-dimethylethan-1-aminium chloride (**14**)

To a suspension of compound **13** (15 mg, 0.039 mmol) in CH_3_OH at 0 °C, a solution of 0.5 M HCl in CH_3_OH (80 μL, 0.039 mmol) was slowly added. The solution was stirred for 2 h at room temperature, then the solvent was evaporated under vacuum to give 16.3 mg of compound **14** as a white solid. Yield: 99%. m.p. 224 °C; ^1^H-NMR (300 MHz, DMSO-*d*_6_), δ: 9.80 (1H, brs); 8.51 (1H, t, J = 5.7 Hz); 8.35 (1H, d, J = 1.7 Hz); 8.02 (1H, dd, J = 1.7, 8.7 Hz); 7.75 (1H, d, J = 8.7 Hz); 7.59 (1H, d, J = 16 Hz); 6.77 (1H, d, J = 16 Hz); 6.36 (1H, s), 6.07 (1H, dd, J = 5.3, 8.7 Hz); 3.74–3.61 (1H, m); 3.60–3.50 (2H, m); 3.44–3.22 (2H, m); 3.23–3.13 (2H, m); 2.37–2.17 (2H, m); 1.77–1.58 (1H, m).

### 3.2. NMR Studies

The ligand stock solution of compound **9** was prepared by dissolving the solid compound in DMSO-d_6_ at 26 mM final concentration. The oligonucleotides were purchased from Eurofins Genomics (Ebersberg, Germany). The NMR samples of duplexes forming self-complementary oligonucleotides 5**′**-d(CGTACG)_2_-3**′** and 5**′**d-(AAGAATTCTT)_2_-3**′** were prepared at 0.30 mM concentration. Both sequences adopt a B-DNA double helix conformation in H_2_O/D_2_O (9:1), 100 mM NaCl and 10 mM sodium phosphate buffer (pH 7.0), as previously determined [40,41]. The ^1^H titration experiments with double helix DNA were performed at 15 °C since the oligonucleotides melt at 25 °C (at the reported salt concentrations). The term d(TTAGGGT)_4_ was used to indicate a tetramolecular G-quadruplex including four d(TTAGGGT). The NMR sample (550 μL) of d(TTAGGGT)_4_ was prepared as a 0.25 mM solution (quadruplex concentration) in H_2_O/D_2_O (9:1) containing 25 mM KH_2_PO_4_, 150 mM KCl and 1 mM EDTA and a pH of 6.7. ^1^H NMR titrations were performed by adding increasing amounts of ligand to the DNA solution. Different ratios of R = [drug]/[DNA] were considered: R = 0, 0.5, 1.0, 1.5, 2.0, etc. (e.g., for R = 0.5, a concentrated ligand solution was added to a 0.30 mM oligonucleotide solution in double helix to obtain a final concentration of the ligand of 0.15 mM). The ^1^H assignments for the free oligonucleotide have been previously reported [48].

The NMR spectra were recorded on a Bruker (Karlsruhe, Germany) AV600 spectrometer operating at a frequency of 600.10 MHz. NMR titrations were performed by adding increasing amounts of ligand to the DNA samples at different ratios of R = [drug]/[DNA]. The protons of duplex oligonucleotides in the complexes with compound **9** were assigned using standard NOESY and TOCSY experiments. Phase-sensitive NOESY spectra of the complexes were acquired at 15 and 25 °C in TPPI mode, with 2048 × 1024 complex FIDs. Mixing times ranged from 150 to 300 ms. TOCSY spectra were acquired with the use of a MLEV-17 spin-lock pulse (60 ms total duration). All spectra were transformed and weighted with a 90° shifted sine-bell squared function to 4K × 4K real data points.

### 3.3. Cytotoxic Assay

The antiproliferative activity was evaluated on human ovarian carcinoma IGROV-1, prostate cancer CW22Rv1 cells and healthy RWPE1 prostate cells. Cells in the logarithmic phase of growth were harvested and seeded in duplicates into 12-well plates. A total of 24 h after seeding, the cells were exposed to the compounds dissolved in DMSO 100% and to cisplatin (Teva, Italia) diluted in saline, used as positive control, for 72 h. The final concentration of DMSO (less than 0,2%) was ineffective on cell growth. The cells were counted with a Coulter counter (Z2 Particle Counter, Beckman Coulter, Milan, Italy). IC_50_ is defined as the inhibitory compound concentration causing a 50% decrease in cell growth over that of the untreated control.

### 3.4. Molecular Modeling Studies

The ligand molecules were refined using a systematic conformer search followed by geometry optimization of the lowest energy structure with MOPAC7 (PM3 Method, RMS gradient 0.0100) [49]. The starting 3D structure of the d(TTAGGGT)_4_ parallel-stranded DNA G-quadruplex was taken from the NMR ensemble deposited in the Protein Data Bank (PDB accession code: 2JWQ) [50] and prepared following the directions previously described [51].

Flexible docking calculations at the d(TTAGGGT)_4_ target were performed with AutoDock 4.2 [52,53] using the Lamarckian genetic algorithm in combination with a grid-based energy evaluation method in order to calculate the grid maps (80Å × 80Å × 80Å box with a spacing of 0.01Å). Gasteiger–Marsili charges [54] were added to the ligand using the AutoDock Toolkit (ADT) [55]. The solvation parameters were added to the system by means of the Addsol utility of AutoDock, and the phosphorus atoms in the G-quadruplex structure were parameterized using the Cornell parameters. The experiment was conducted with an initial population consisting of 100 randomly placed Escholidine molecules. The maximum number of energy evaluations was set at 250 with an elitism value of 1, a mutation rate of 0.02 and a crossover rate of 0.80. The local search was conducted using 250 independent docking runs using the pseudo-Solis–Wets algorithm, with a maximum of 250 iterations per local search. The docking results were scored using an in-house version of the simpler intermolecular energy function based on the Weiner force field, and the lowest energy conformations (differing by less than 1.0Å in positional root-mean-square deviation (rmsd)) were collected.

## 4. Conclusions

Kynurenic acid (KYNA) and derivatives have recently been shown to have a role in carcinogenesis and cancer therapy. In this study, we investigated compounds structurally related to KYNA which were synthesized with the aim of obtaining new G-quadruplex DNA binders. The specific DNA binding mode of a selected analogue (**9**) with two models of duplex DNA—5**′**-d(CGTACG)_2_-3**′** and 5**′**-d(AAGAATTCTT)_2_-3**′** oligonucleotides—was carried out. The findings allowed for the exclusion of an intercalation of compound **9** with both oligonucleotides, but suggested a slight association by an external binding mode at the level of AT base pairs. On the other hand, the experiments carried out with d(TTAGGGT)_4_, a model of human telomere sequence, suggested that compound **9** binds the G-quadruplex in an intermediate regime on the NMR time scale. Furthermore, the compound is able to interact with the aromatic ring at the 3**′**-end tetrad (G6), though without efficient stacking. A three-dimensional model of the top-ranked complex between compound **9** and the d(TTAGGGT)_4_ parallel-stranded DNA G-quadruplex, as obtained from the molecular docking experiment, showed that the ligand fits between the G6 tetrad and T7 of the d(TTAGGGT)_4_ G-quadruplex. The side chain of compound **9** orients along the groove, with the quaternary nitrogen forming two salt bridges with G5OP_2_ and G6OP_2_, contributing to the stabilization of the complex. In conclusion, we have identified a new nature-inspired scaffold which can be considered a starting point for the development of potential G-quadruplex stabilizing molecules. Overall, these results do not provide evidence of a strong G-quadruplex interaction, but they are a further confirmation of the crucial role of a planar system and one (or more) charged nitrogen-containing groups in facilitating the binding of small molecules to G-quadruplex grooves and loops. Furthermore, the presence of a flexible and modulable chain connecting the amino group to the polycyclic core could allow for a better allocation of the molecule inside the G-quadruplex structure with an implemented interaction and stabilization of the complex.

## Figures and Tables

**Table 1:** Cytotoxic activity of kynurenic acid derivatives on human cell lines ^a^. Cisplatin was used as positive control.

Compound	Cell Line (IC_50_ μM)	
	IGROV-1	RWPE1
Cisplatin	0.38 ± 0.049	-
**9**	14.5 ± 5	>100
**10**	>30	-
**14**	>30	>100

^a^ After 72 h exposure, the cells were harvested and counted with a Coulter counter.

## Data Availability

Not applicable.

## Supplementary Materials

The following supporting information can be downloaded at: https://www.mdpi.com/article/10.3390/molecules27092791/s1, Table S1: ^1^H NMR chemical shift values relative to compound **9** with duplexes. Table S2: ^1^H NMR chemical shift values relative to compound **9** with quadruplex. Figure S1: NOESY spectrum of compound **9** in H_2_O/D_2_O (9:1). Figure S2: Aromatic and anomeric proton region of NOESY spectrum of 5′-d(CGTACG)_2_-3′ with compound **9**. Figure S3: Aromatic proton region of NOESY spectrum of 5′d-(AAGAATTCTT)_2_-3′ with compound **9**. Figure S4: Imino and aromatic proton region of NOESY spectrum of d(TTAGGGT)_4_ with compound **9**. ^1^H-NMR and ^13^C-NMR spectra of synthesized compounds.

## Sample Availability

Samples of the compounds are available from the authors.
