# Supplementary material for: Synthesis and Investigation of the G-Quadruplex Binding Properties of Kynurenic Acid Derivatives with a Dihydroimidazoquinoline-3,5-dione Core
Source: Molecules. 2022 Apr 27;27(9):2791. doi: 10.3390/molecules27092791 (PMC9103425; doi:10.3390/molecules27092791)
Supplement: Supplementary file 1 [file molecules-27-02791-s001.zip › molecules-1665029-supplementary.pdf]

Synthesis and investigation of G-quadruplex binding properties of Kynurenic acid derivatives with a dihydroimidazoquinoline-3,5-dione core

Stefania Mazzini, Salvatore Princiotto *, Loana Musso, Daniele Passarella, Giovanni Luca Beretta, Paola Perego, Sabrina Dallavalle

Department of Food, Environmental and Nutritional Sciences (DeFENS), University of Milan, Via Celoria 2, 20133 Milan, Italy; stefania.mazzini@unimi.it (S.M.); salvatore.princiotto@unimi.it (S.P.); loana.musso@unimi.it (L.M.); sabrina.dallavalle@unimi.it (S.D.)
Department of Chemistry, University of Milan, 20133 Milan, Italy; daniele.passarella@unimi.it
Molecular Pharmacology Unit, Department of Applied Research and Technological Development, Fondazione IRCCS Istituto Nazionale Tumori, Via Amadeo 42, 20133 Milan, Italy; giovanni.beretta@istitutotumori.mi.it (G.L.B.);paola.perego@istitutotumori.mi.it (P.P.)

Supplementary Material

Table of contents

Table S1. Selected ^{1}H NMR chemical shift values for the complex of **9** with 5′-d(CGTACG)$_2$-3′ and 5′-d(AAGAATTCTT)$_2$-3′ duplexes. **S2**

Table S2. Selected ^{1}H chemical shift values for the complex of **9** with d(TTAGGGT)$_4$. **S3**

Figure S1: NOESY spectrum of **9** in H_2O/D_2O (9:1), 100 mM NaCl, 10 mM sodium phosphate buffer (pH 7.0), T = 25 °C. **S3**

Figure S2. Aromatic and anomeric protons region of NOESY spectrum of 5′-d(CGTACG)$_2$-3′ with **9**, t_{mix} = 300 ms, at 15 °C in H_2O/D_2O (9:1), 100 mM NaCl, 10 mM sodium phosphate buffer (pH 7.0), at R = [**9**]/[DNA] = 2.0. The arrows indicate the broad proton signals of compound **9**. **S4**

Figure S3. Aromatic protons region of NOESY spectrum of 5′d-(AAGAATTCTT)$_2$-3′ with **9**, t_{mix}=300ms at 15 °C in H_2O/D_2O (9:1), 100 mM NaCl, 10 mM sodium phosphate buffer (pH 7.0), at R = [**9**]/[DNA] = 2.0. The arrows indicate the broad proton signals of compound **9**. **S4**

Figure S4. Imino and aromatic proton region of NOESY spectrum of d(TTAGGGT)$_4$ with **9**, t_{mix}=300ms, at 25 °C in H_2O/D_2O (9:1), 150 mM KCl, 25 mM K^+ phosphate buffer, EDTA 1 mM (pH 6.7), at R = [**9**]/[DNA] = 4.0. The arrows indicate the broad proton signals of **9**. **S5**

^{1}H-NMR and ^{13}C-NMR spectra of synthesized compounds **S6-S15**

Table S1. Selected ^{1}H NMR chemical shift values for the complex of **9** with 5′-d(CGTACG)$_2$-3′ and 5′-d(AAGAATTCTT)$_2$-3′ duplexes[a,b].

6-mer	H2/H5/CH_3	Δδ[c]	H6/H8	Δδ[c]	H1′	Δδ[c]
C1	5.90	0.04	7.68	-0.02	5.76	0.00
G2	-	-	8.03	0.00	5.99	-0.04
T3	1.57	-0.01	7.31	-0.01	5.65	-0.05
A4	7.59	-0.09	8.38	+0.03	6.22	-0.08
C5	5.39	-0.02	7.31	-0.01	5.65	0.00
G6	-	-	7.92	0.00	6.11	0.00
	NH					
C1G6	-	-				
G2C5	12.76	-0.18				
T3A4	13.60	-0.07				

10-mer	H2/H5/CH_3	Δδ[c]	H6/H8	Δδ[c]
A1	-	-	7.81	-0.20
A2	-	-	8.10	0.00
G3	-	-	7.69	-0.01
A4	7.25	+0.03	8.10	+0.02
A5	7.69	-0.09	8.10	0.00
T6	1.27	-0.03	7.10	0.00
T7	1.54	-0.04	7.39	-0.05
C8	5.63	-0.05	7.69	-0.06
T9	1.73	-0.07	7.50	-0.09
T10	1.73	-0.08	7.50	-0.13
	NH			
G3C8	12.32	-0.08		
A4T7	13.65	-0.10		
A5T6	13.58	-0.10		

[a]Measured at 15 °C in H_2O/D_2O (9:1), 100 mM NaCl, 10 mM sodium phosphate buffer (pH 7.0) and R = 2.0.
[b]Measured in ppm from external DSS.
[c]$\Delta\delta = \delta_{bound} - \delta_{free}$.

Table S2. Selected ^{1}H chemical shift values for the complex of **9** with d(TTAGGGT)$_4$[a]

	complex with **9**			
	H1/H2/Me	Δδ[b]	H6/H8	Δδ
T1	n.d.	-	7.55	+0.05
T2	1.83	+ 0.08	7.37	+0.06
A3	8.09	- 0.08	8.44	+0.05
G4	11.52	- 0.09	7.85	-0.09
G5	11.15	- 0.07	7.68	- 0.05
G6	10.80	- 0.21	7.71	- 0.05
T7	1.75	+0.15	7.54	+0.25

[a]Measured at 25 °C in ppm (δ) from external DSS. Solvent H_2O-D_2O (90:10 v/v), 25 mM K^+ phosphate buffer, 150 mM KCl, 1 mM EDTA, pH 6.7. *R* = [ligand] / [DNA] = 2.0. T1 signals were not detected. The ribose protons showing significant shift variations are: T2H1′ Δδ = +0.28; T7H1′ Δδ = +0.14.
[b]$\Delta\delta = \delta_{bound} - \delta_{free}$.

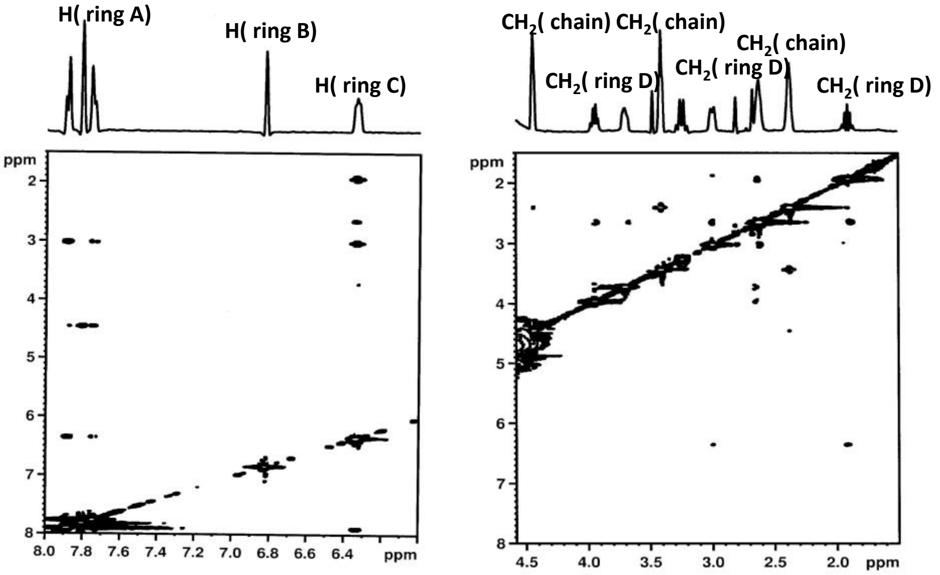

Figure S1: NOESY spectrum of **9** in H_2O/D_2O (9:1), 100 mM NaCl, 10 mM sodium phosphate buffer (pH 7.0), T = 25 °C.

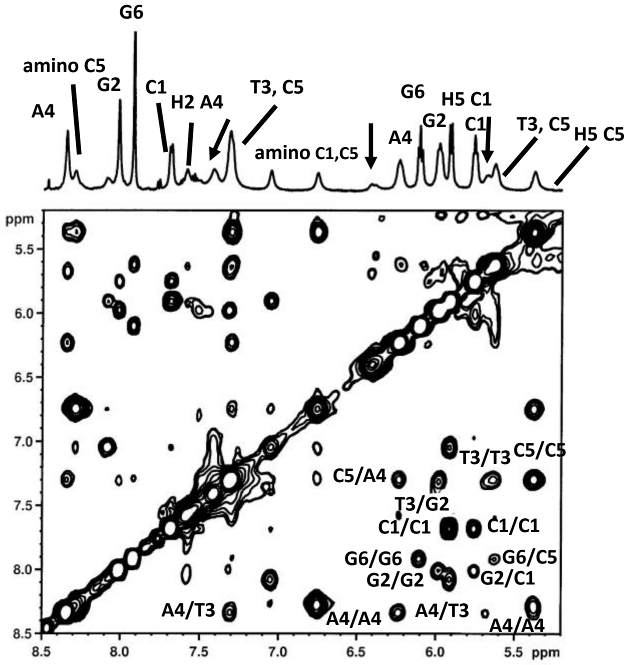

Figure S2. Aromatic and anomeric protons region of NOESY spectrum of 5′-d(CGTACG)$_2$-3′ with **9**, t_{mix} = 300 ms, at 15 °C in H_2O/D_2O (9:1), 100 mM NaCl, 10 mM sodium phosphate buffer (pH 7.0), at R = [**9**]/[DNA] = 2.0. The arrows indicate the broad proton signals of compound **9**.

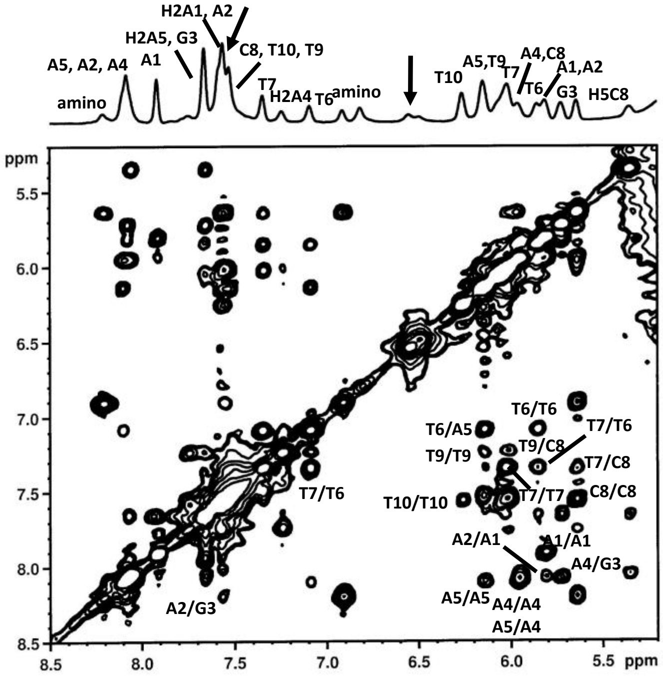

Figure S3. Aromatic protons region of NOESY spectrum of 5′d-(AAGAATTCTT)$_2$-3′ with **9**, t_{mix} = 300 ms at 15 °C in H_2O/D_2O (9:1), 100 mM NaCl, 10 mM sodium phosphate buffer (pH 7.0), at R = [**9**]/[DNA] = 2.0. The arrows indicate the broad proton signals of compound **9**.

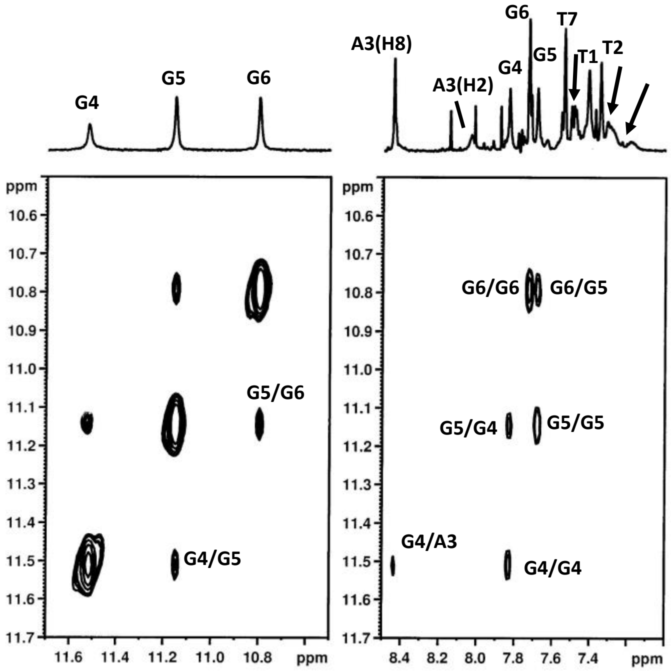

Figure S4. Imino and aromatic protons region of NOESY spectrum of d(TTAGGGT)$_4$ with **9**, t_{mix} = 300 ms, at 25 °C in H_2O/D_2O (9:1), 150 mM KCl, 25 mM K^+ phosphate buffer, EDTA 1 mM (pH 6.7), at R = [**9**]/[DNA] = 4.0. The arrows indicate the broad proton signals of **9**.

^{1}H-NMR of compound **4a** in DMSO-*d*$_6$

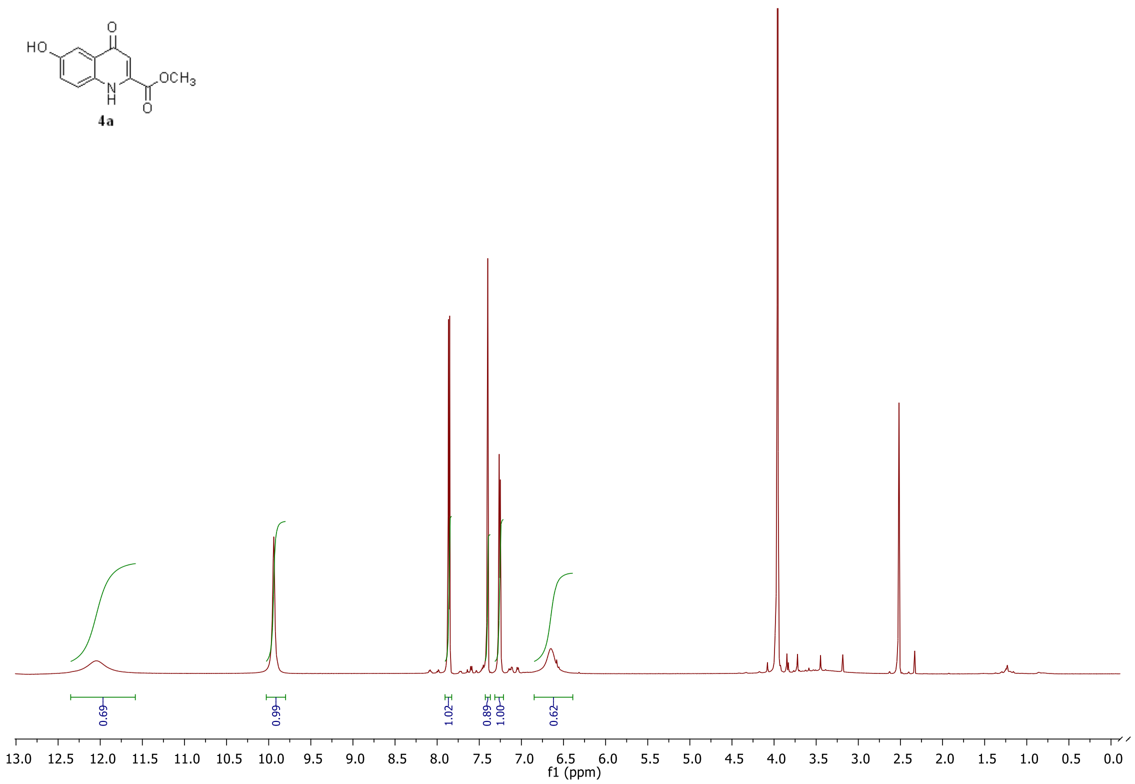

^{13}C-NMR of compound **4a** in DMSO-*d*$_6$

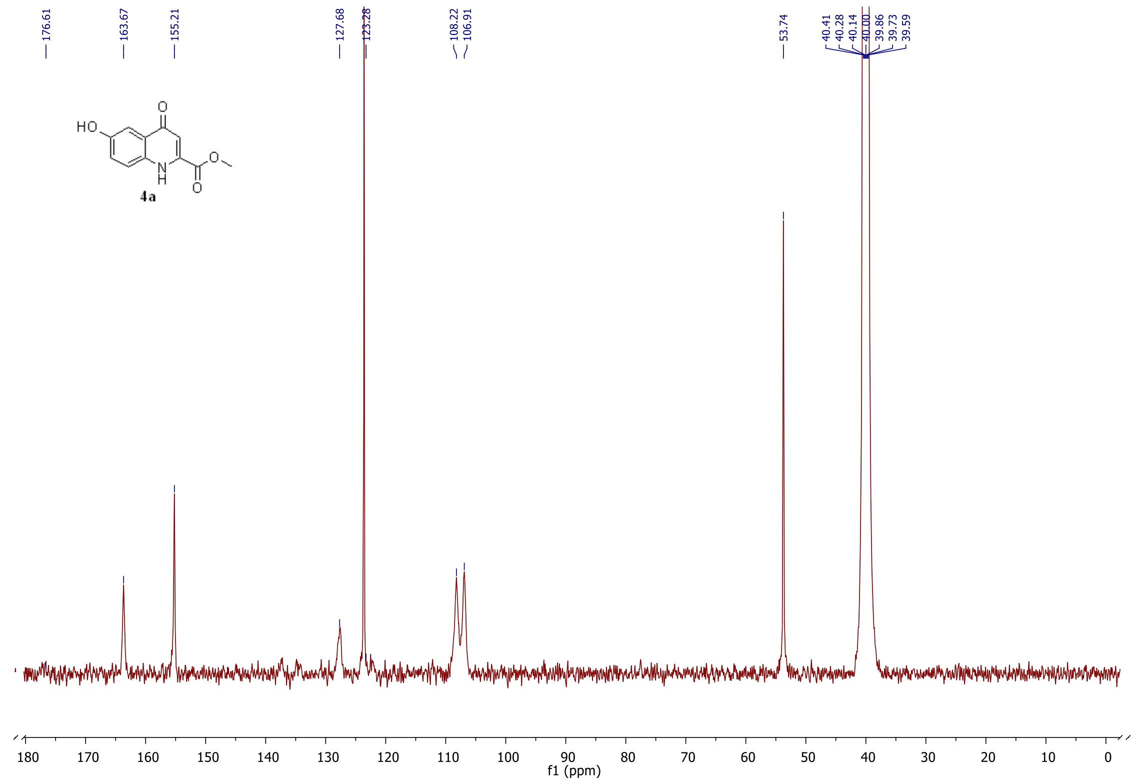

^{1}H-NMR of compound **5a** in DMSO-*d*6

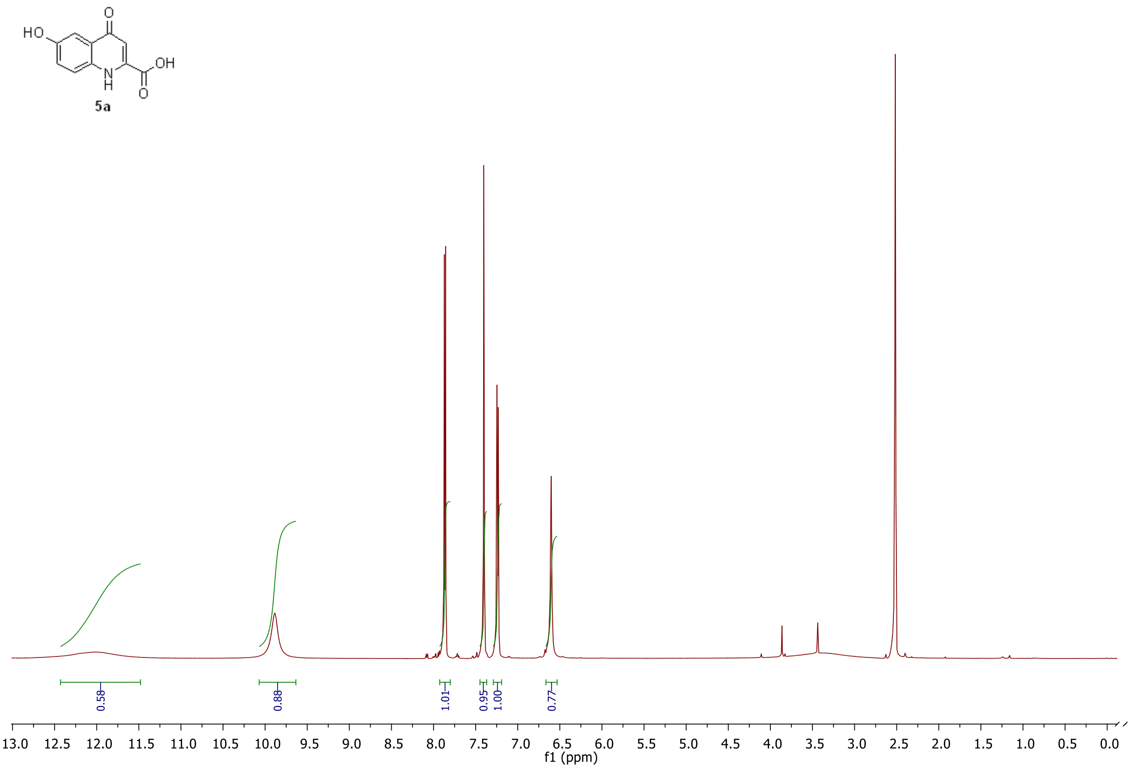

^{13}C-NMR of compound **5a** in DMSO-*d*6

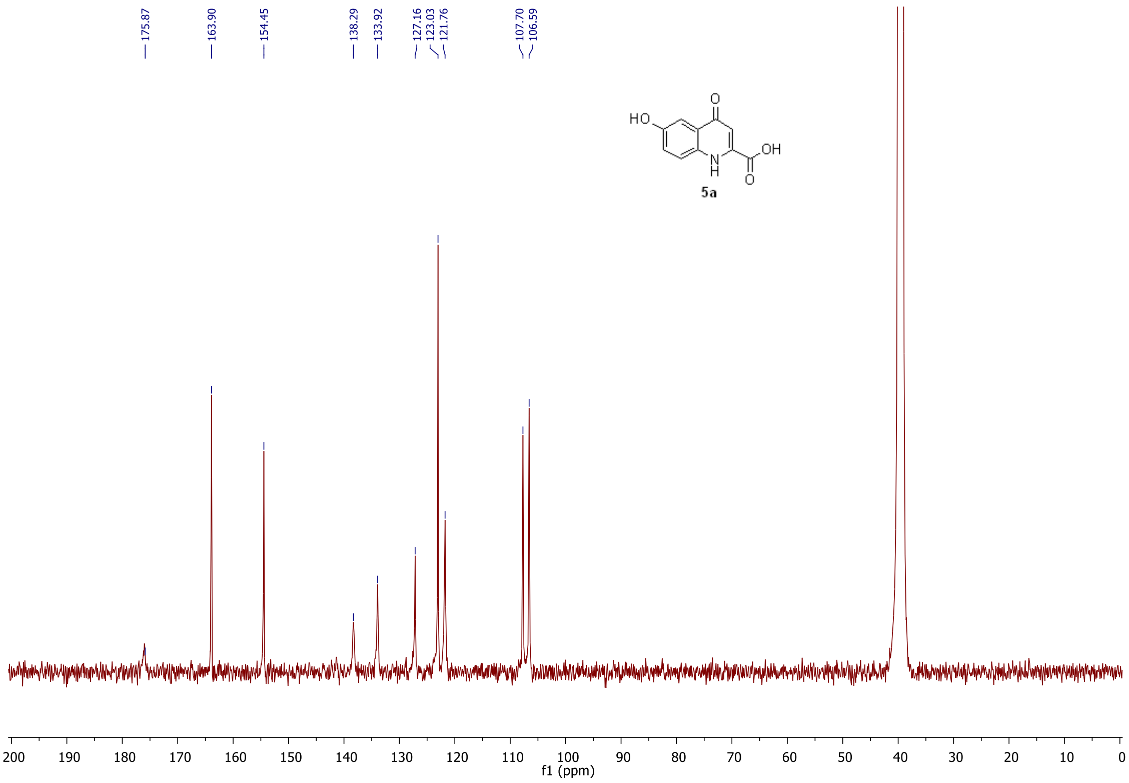

^{1}H-NMR of compound **6a** in DMSO-*d*6

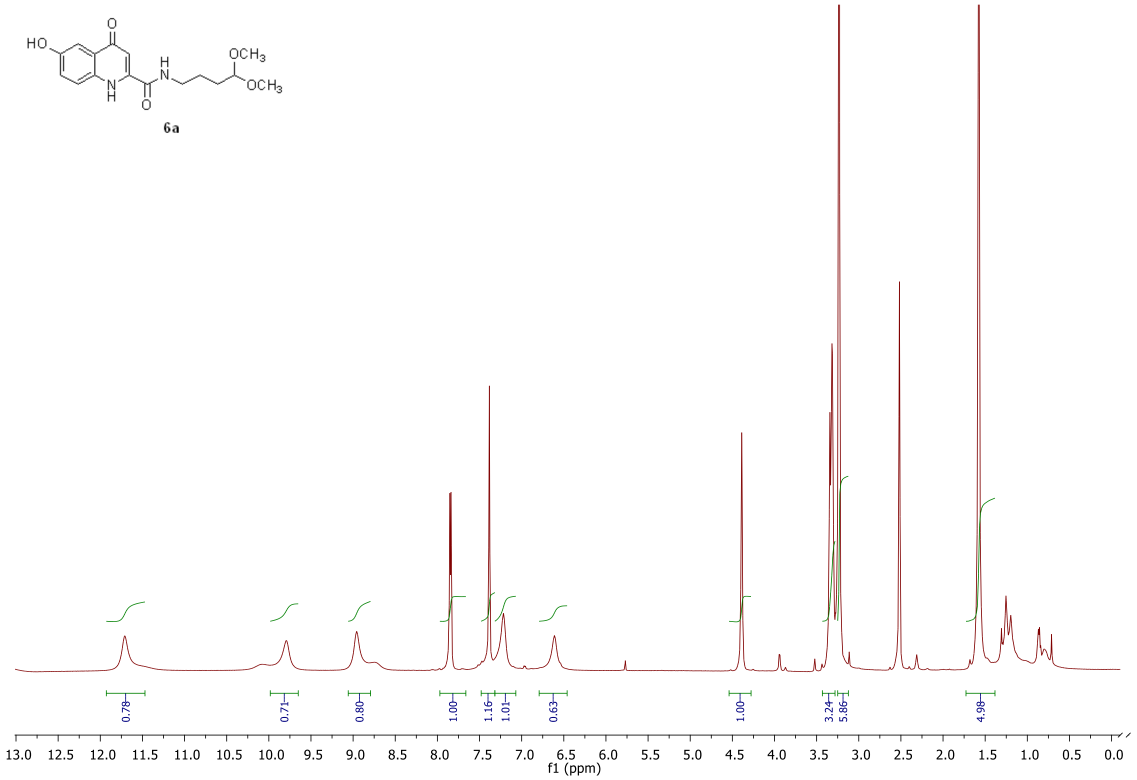

^{13}C-NMR of compound **6a** in DMSO-*d*6

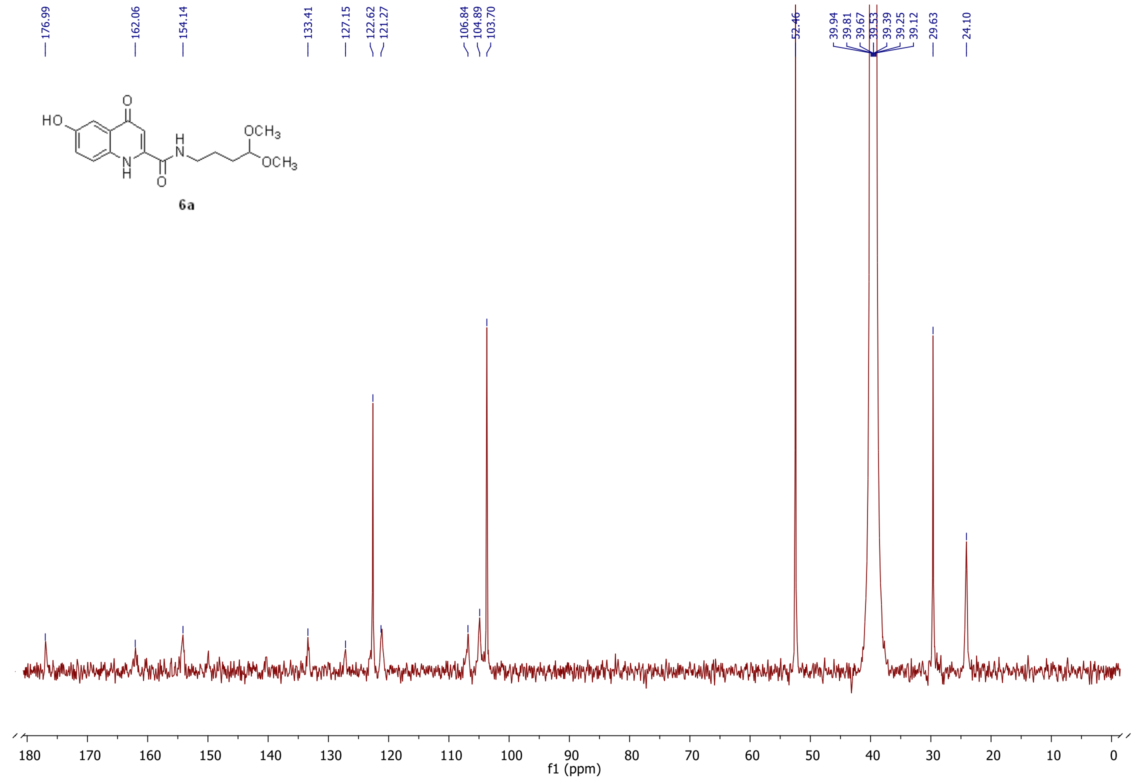

^{1}H-NMR of compound **7a** in DMSO-d_6

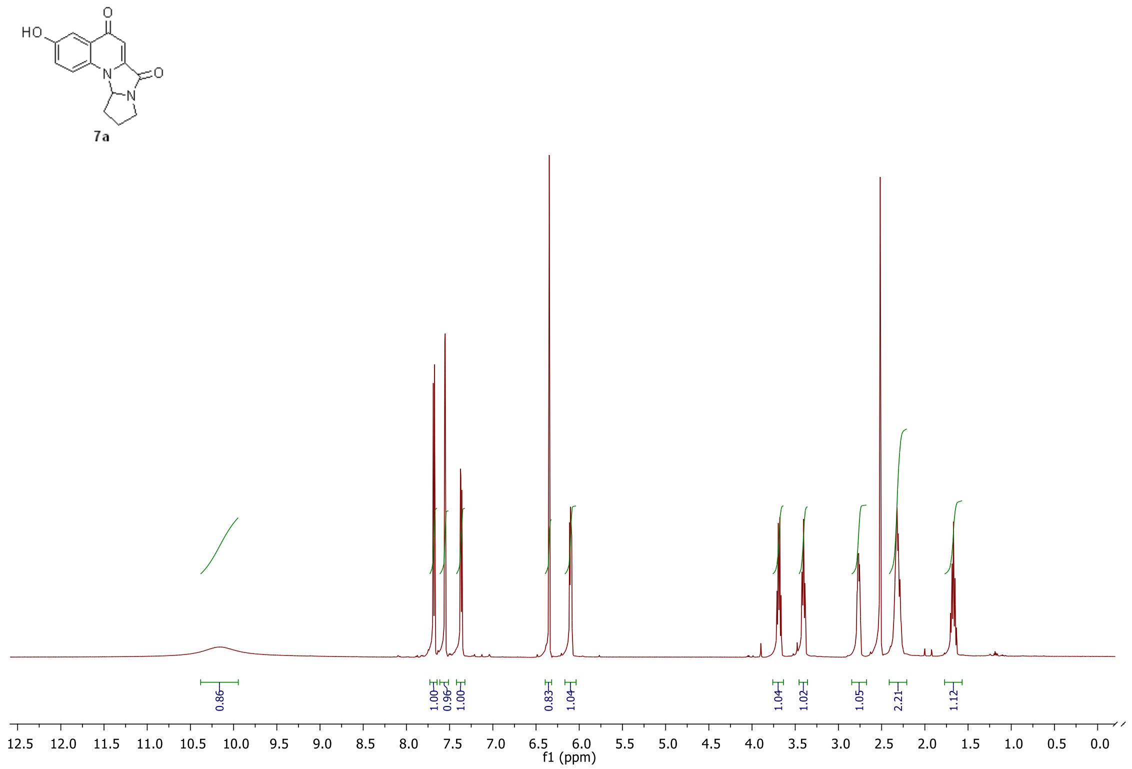

^{13}C-NMR of compound **7a** in DMSO-d_6

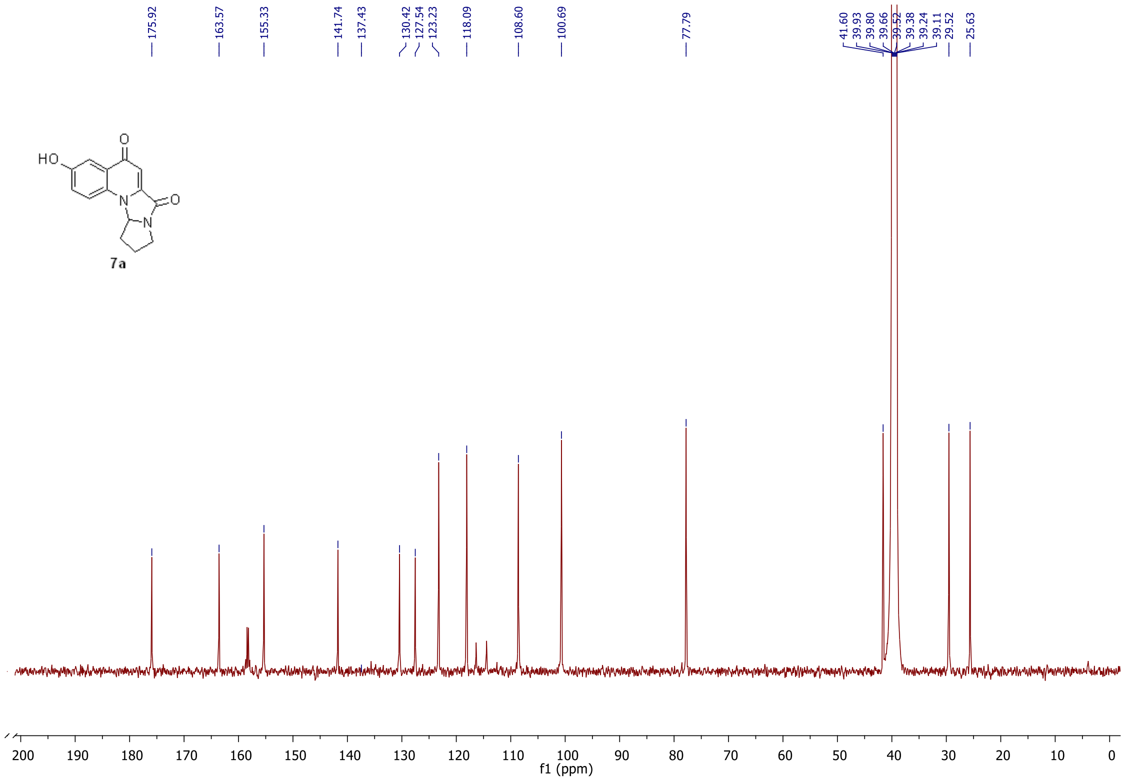

NMR spectra of compounds **6b** and **7b** are reported in R. Cincinelli et al. *Tetrahedron 70* (**2014**) 9797-9804.

^{1}H-NMR of compound **8** in DMSO-d_6

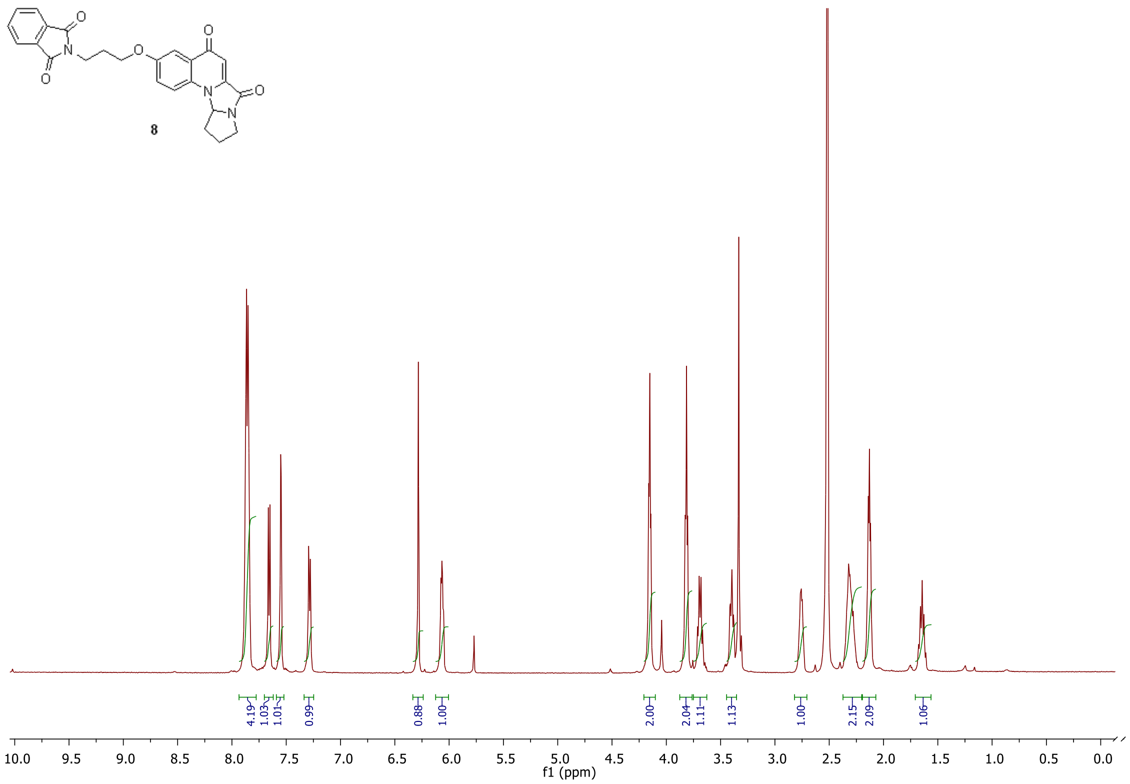

^{13}C-NMR of compound **8** in DMSO-d_6

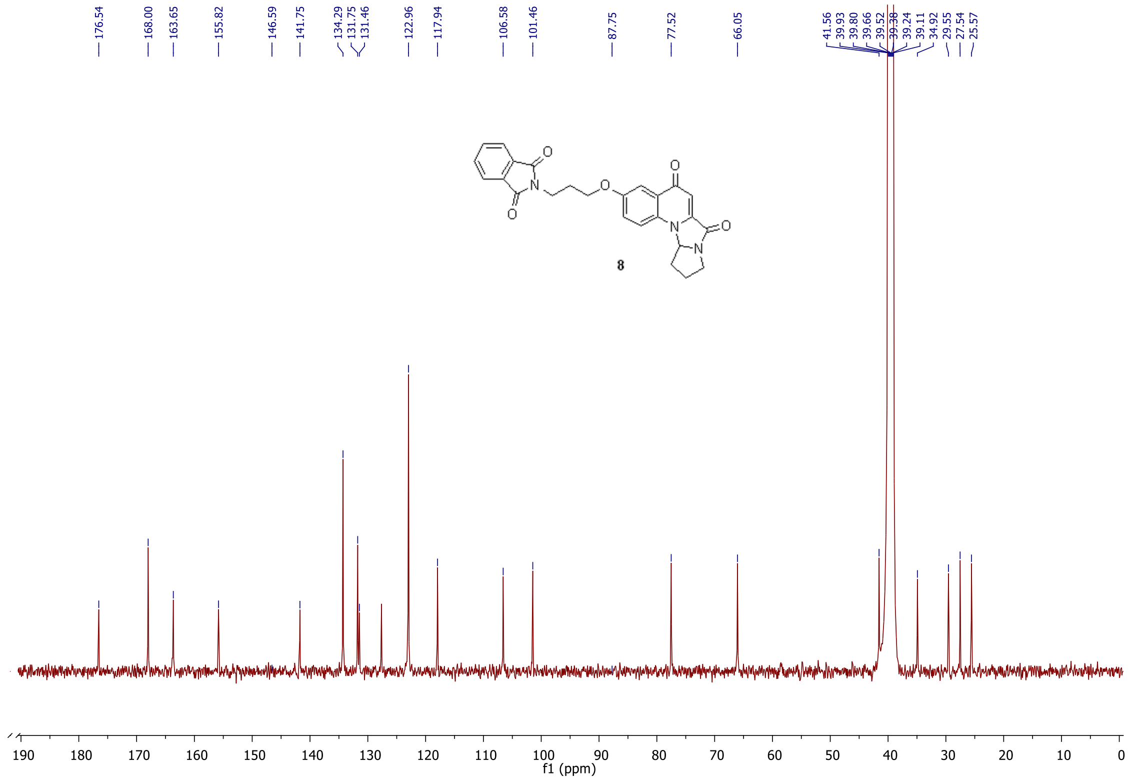

^{1}H-NMR of compound **9** in CH_3OH-d_4

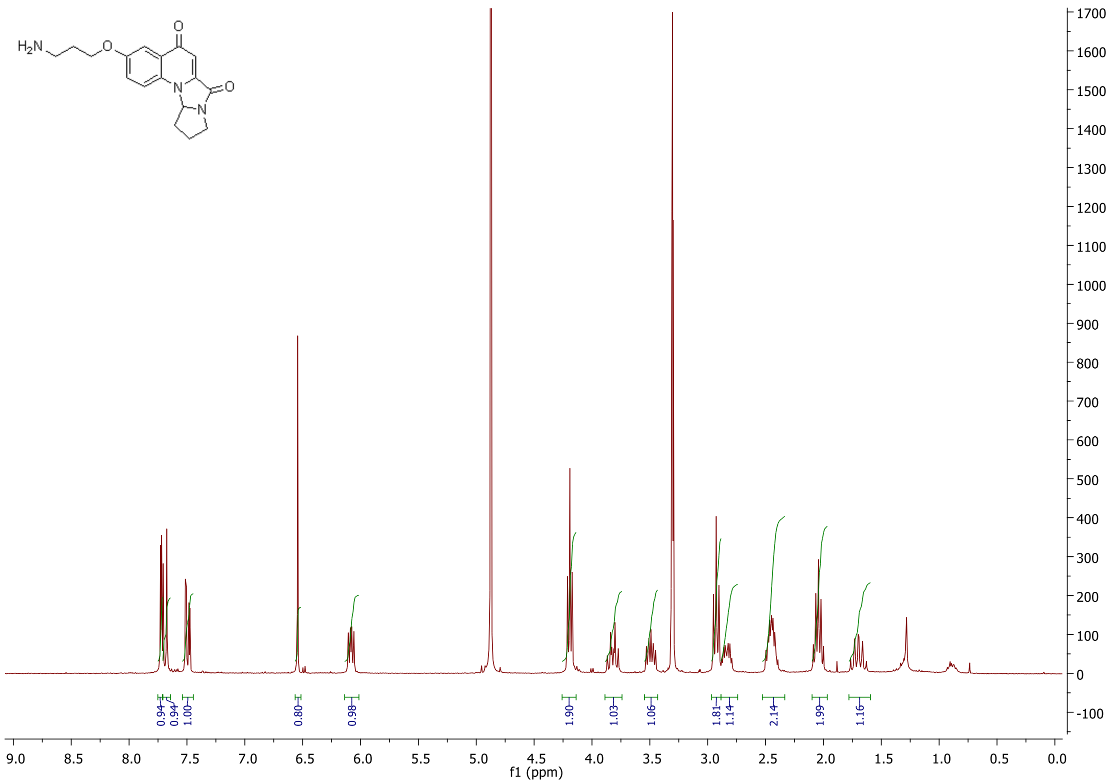

^{13}C-NMR of compound **9** in DMSO-d_6.

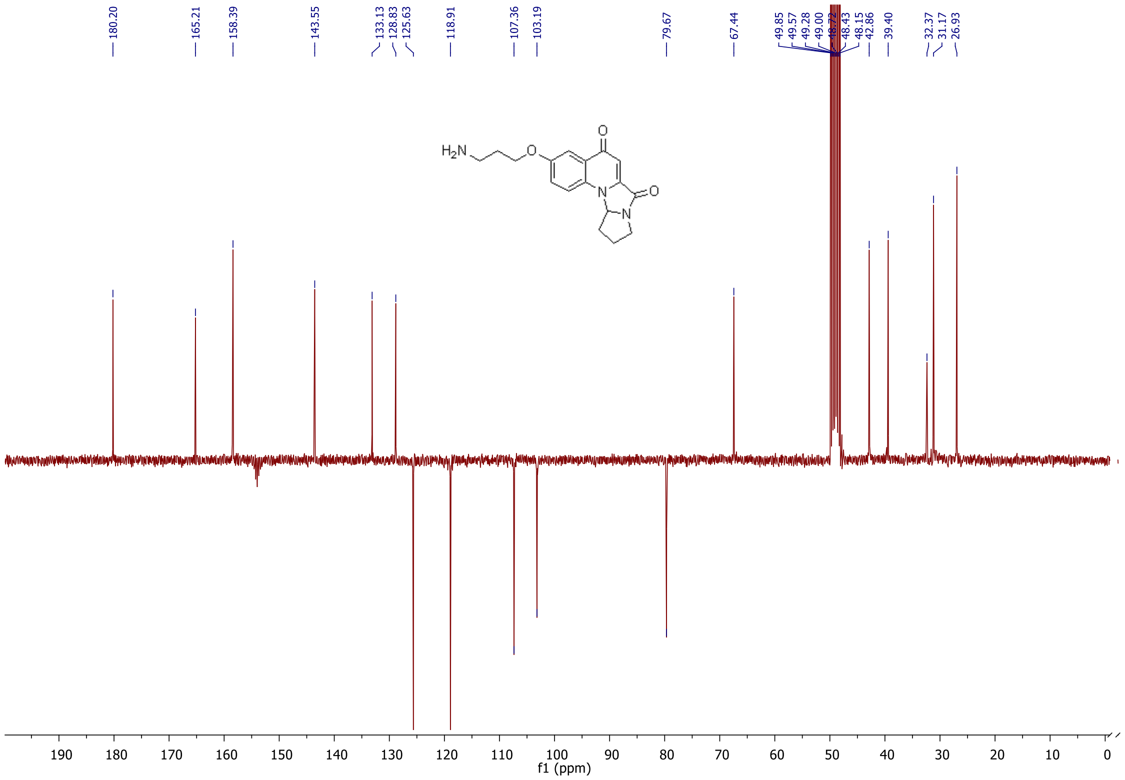

^{1}H-NMR of compound **10** in DMSO-d_6.

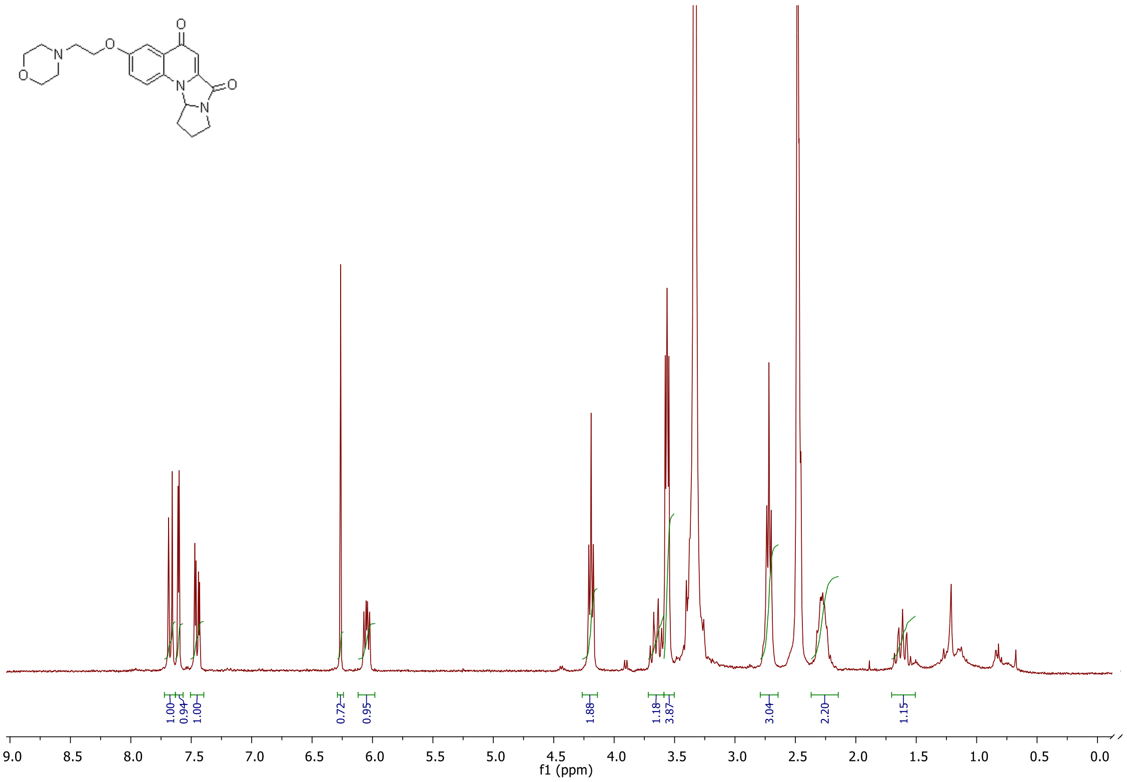

^{13}C-NMR of compound **10** in DMSO-d_6

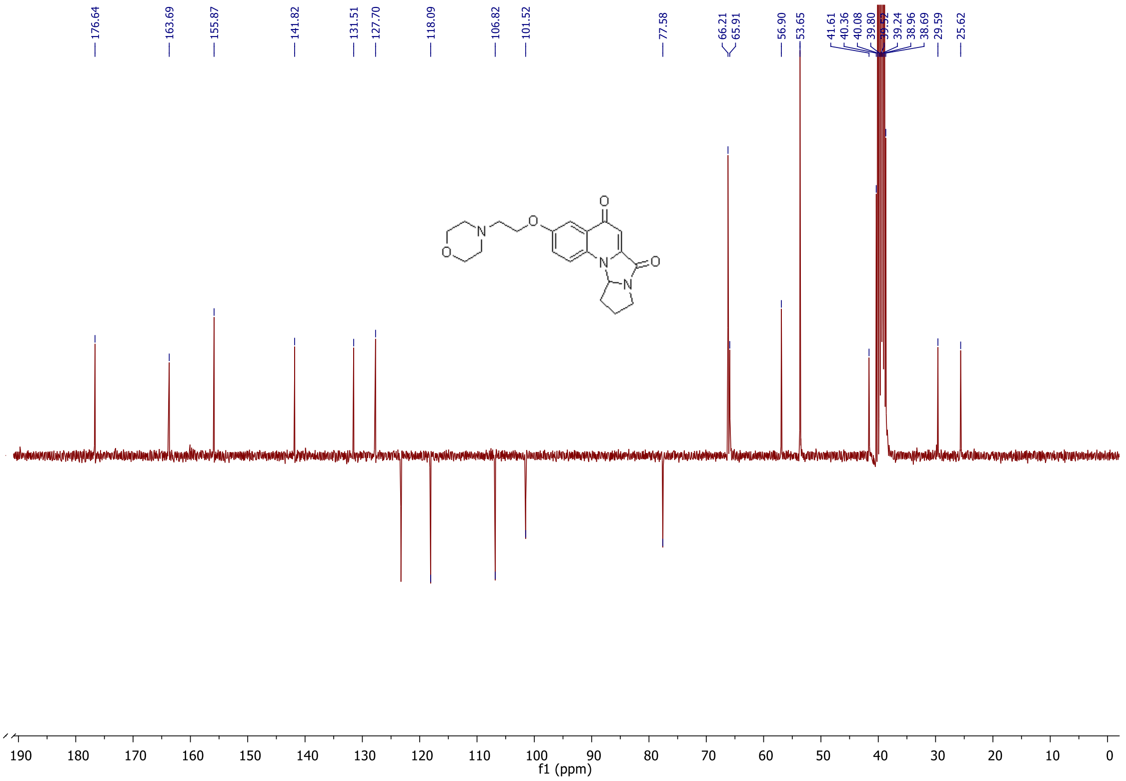

^{1}H-NMR of compound **11** in $CDCl_3$

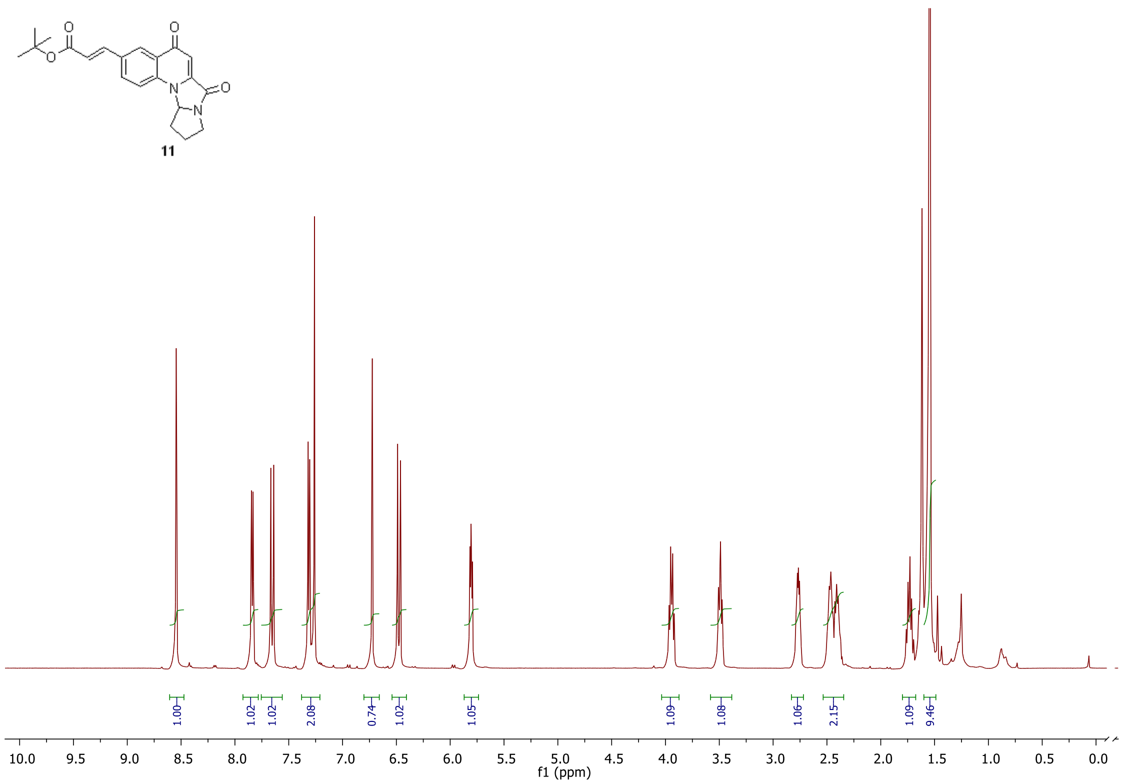

^{13}C-NMR of compound **11** in $CDCl_3$

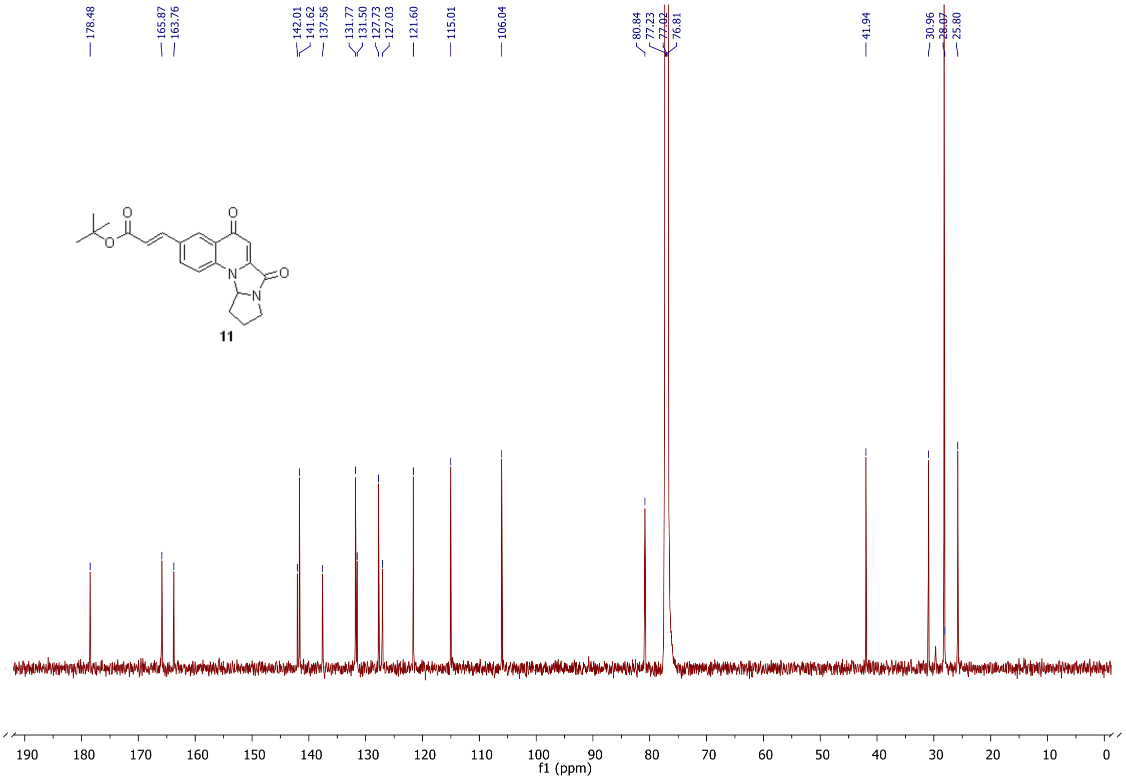

^{1}H-NMR of compound **12** in DMSO-*d*$_6$

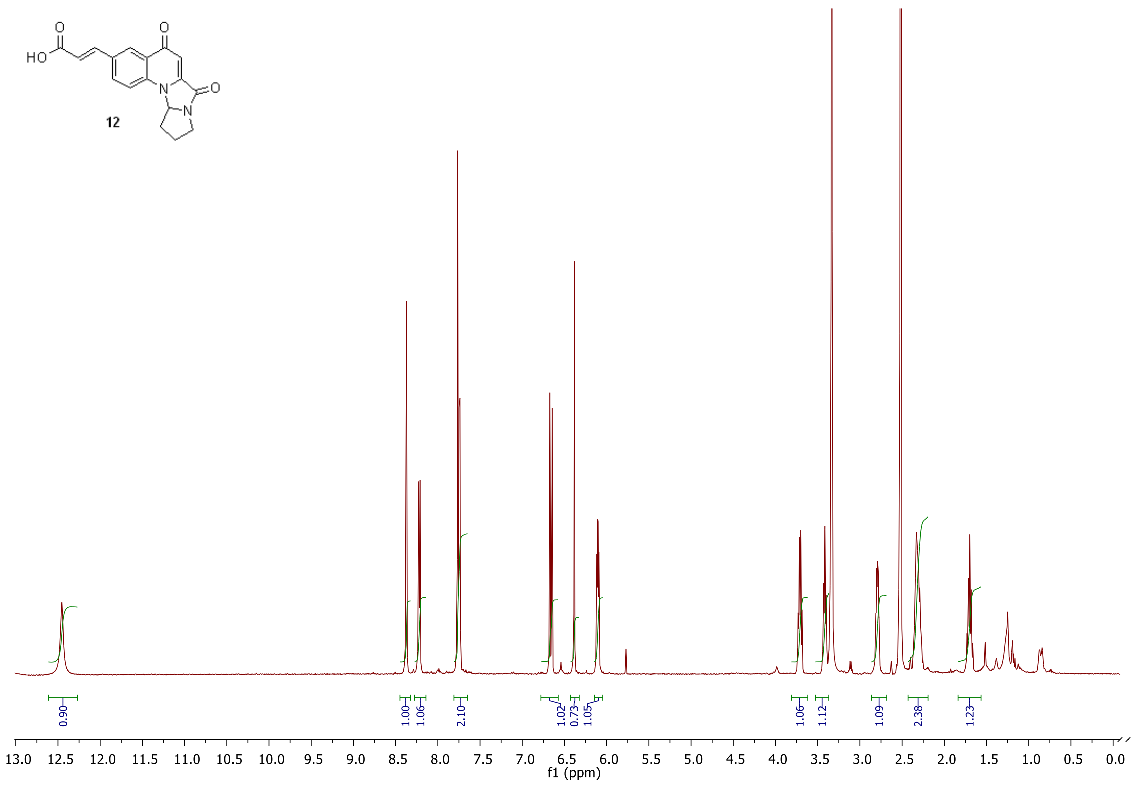

^{13}C-NMR of compound **12** in DMSO-*d*$_6$

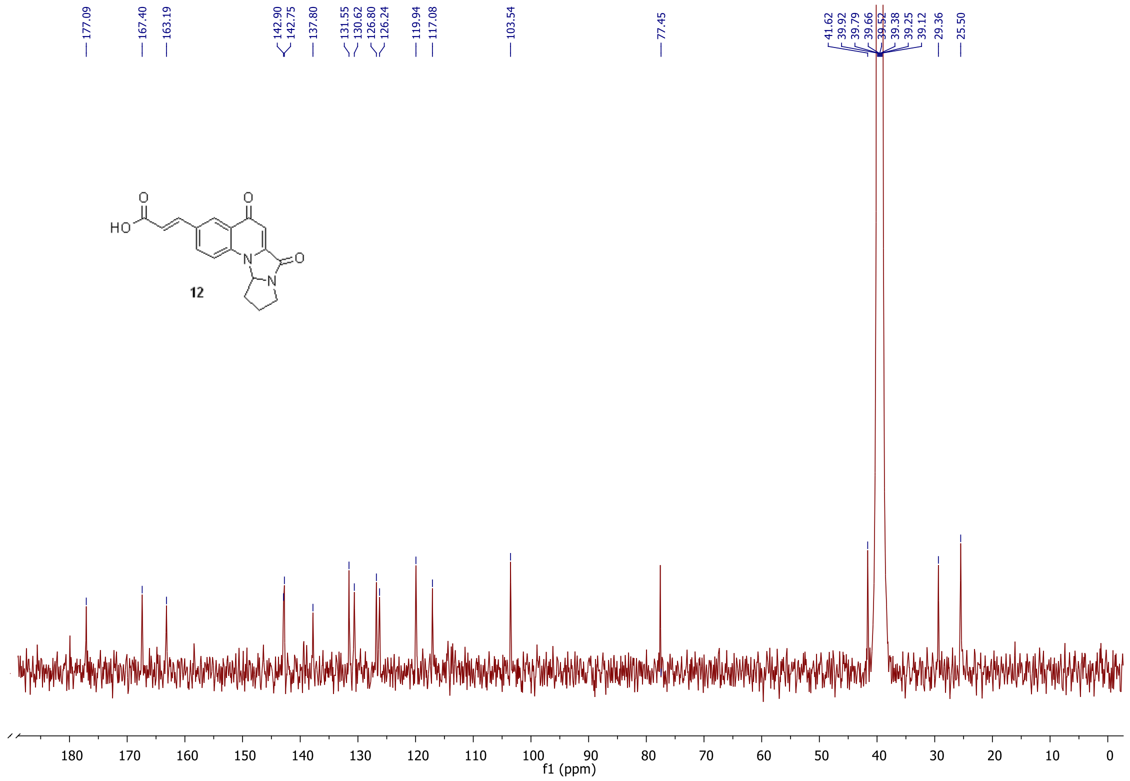

^{1}H-NMR of compound **13** in DMSO-d_6

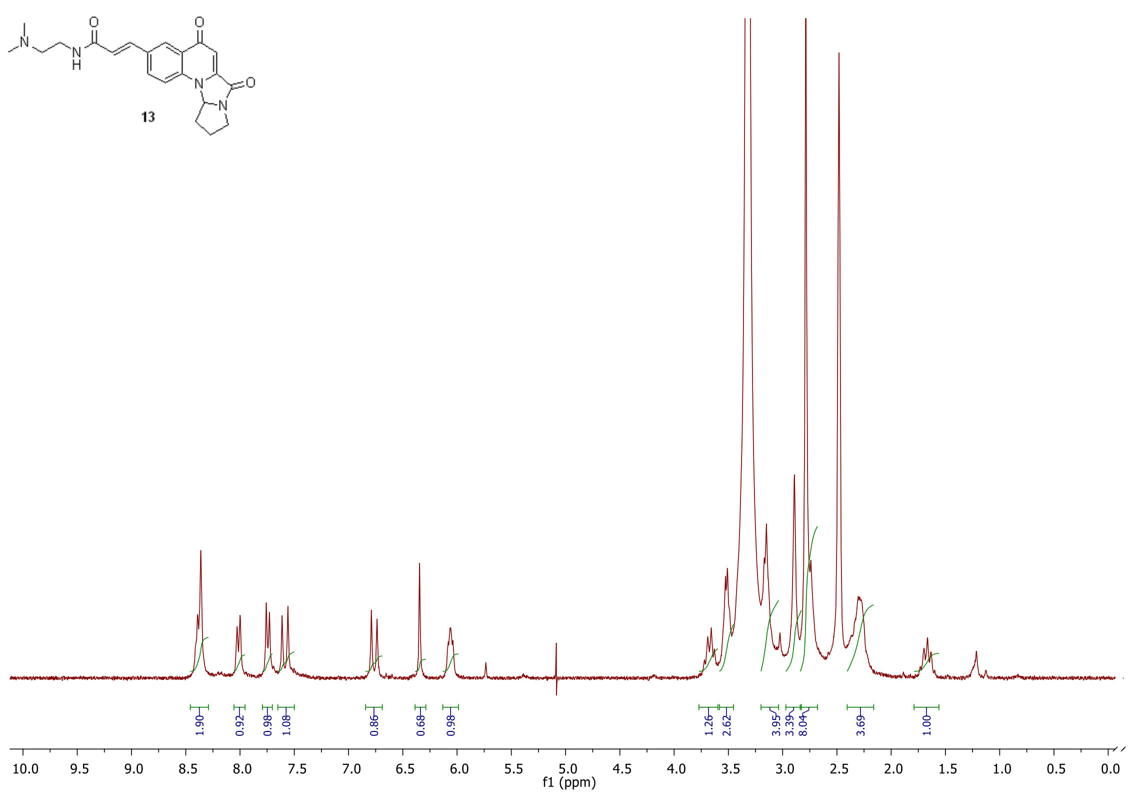

^{13}C-NMR of compound **13** in DMSO-d_6

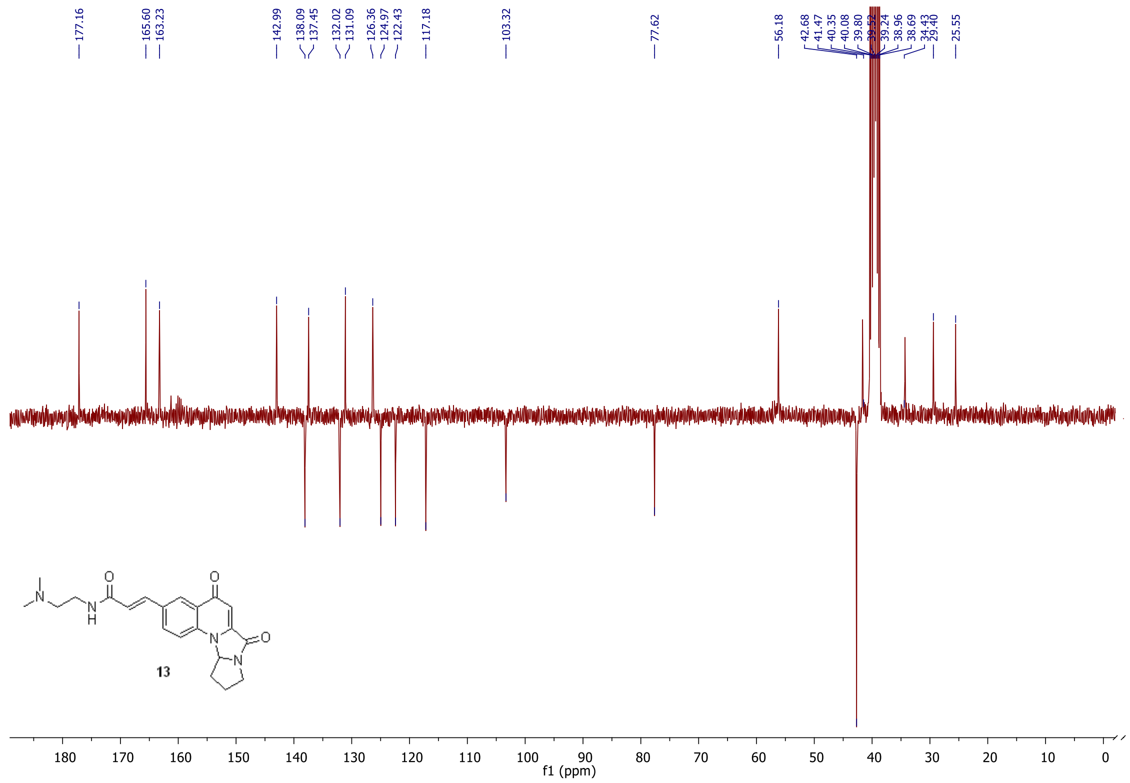

The hydrochloric salts **9**, **10**, and **14** were prepared by treatment of the corresponding free bases with 0.5 N HCl in methanol.
